# Association of lysophosphatidic acids with cerebrospinal fluid biomarkers and progression to Alzheimer’s disease

**DOI:** 10.1186/s13195-020-00680-9

**Published:** 2020-10-02

**Authors:** Shahzad Ahmad, Adelina Orellana, Isabelle Kohler, Lutz Frölich, Itziar de Rojas, Silvia Gil, Mercè Boada, Isabel Hernández, Lucrezia Hausner, Margot H. M. Bakker, Alfredo Cabrera-Socorro, Najaf Amin, Alfredo Ramírez, Agustín Ruiz, Thomas Hankemeier, Cornelia M. Van Duijn

**Affiliations:** 1 Department of Epidemiology, Erasmus Medical Centre, Rotterdam, The Netherlands; 2 Research Center and Memory Clinic Fundació ACE, Institut Català de Neurociències, Aplicades. Universitat Internacional de Catalunya, Barcelona, Spain; 3 Centro de Investigación Biomédica en Red de Enfermedades Neurodegenerativas (CIBERNED), Instituto de Salud Carlos III, Madrid, Spain; 4 Division of Systems Biomedicine and Pharmacology, Leiden Academic Centre for Drug Research, Leiden University, Leiden, The Netherlands; 5 Department of Geriatric Psychiatry, Central Institute of Mental Health, Medical Faculty Mannheim, University of Heidelberg, 68159 Mannheim, Germany; 6 Institute of Cognitive and Clinical Neuroscience, Central Institute of Mental Health, Medical Faculty Mannheim, Heidelberg University, 68159 Mannheim, Germany; 7 Discovery Research, AbbVie Deutschland GmbH & Co. KG, Knollstrasse, 67061 Ludwigshafen, Germany; 8 Janssen Pharmaceutical NV, Turnhoutseweg 30, 2340 Beerse, Belgium; 9 Department for Neurodegenerative Diseases and Geriatric Psychiatry, University of Bonn, Bonn, Germany; 10 Division of Neurogenetics and Molecular Psychiatry, Department of Psychiatry and Psychotherapy, Medical Faculty, University of Cologne, Cologne, Germany; 11 Nuffield Department of Population Health, Oxford University, Oxford, UK

**Keywords:** Lysophosphatidic acids, Pro-inflammatory phospholipids, Signaling lipids, CSF biomarkers, Alzheimer’s disease, MCI

## Abstract

**Background:**

Lysophosphatidic acids (LPAs) are bioactive signaling phospholipids that have been implicated in Alzheimer’s disease (AD). It is largely unknown whether LPAs are associated with AD pathology and progression from mild cognitive impairment (MCI) to AD.

**Methods:**

The current study was performed on cerebrospinal fluid (CSF) and plasma samples of 182 MCI patients from two independent cohorts. We profiled LPA-derived metabolites using liquid chromatography-mass spectrometry. We evaluated the association of LPAs with CSF biomarkers of AD, Aβ-42*,* p-tau, and total tau levels overall and stratified by *APOE* genotype and with MCI to AD progression.

**Results:**

Five LPAs (C16:0, C16:1, C22:4, C22:6, and isomer-LPA C22:5) showed significant positive association with CSF biomarkers of AD, Aβ-42*,* p-tau, and total tau, while LPA C14:0 and C20:1 associated only with Aβ-42 and alkyl-LPA C18:1, and LPA C20:1 associated with tau pathology biomarkers. Association of cyclic-LPA C16:0 and two LPAs (C20:4, C22:4) with Aβ-42 levels was found only in *APOE* ε4 carriers. Furthermore, LPA C16:0 and C16:1 also showed association with MCI to AD dementia progression, but results did not replicate in an independent cohort.

**Conclusions:**

Our findings provide evidence that LPAs may contribute to early AD pathogenesis. Future studies are needed to determine whether LPAs play a role in upstream of AD pathology or are downstream markers of neurodegeneration.

## Background

Lipids play a key role in Alzheimer’s disease (AD) [1–3]. Lysophosphatidic acids (LPAs) are bioactive phospholipids representing a significant class of signaling molecules [4]. LPAs regulate a plethora of downstream processes including brain immune response [5], myelination [6], synaptic transmission [7, 8], and synaptic plasticity [9], as well as in endothelial cells and neurovascular function [10]. A recent study has reported altered levels of LPA C18:2 in AD patients compared to controls in plasma [11] and LPAs have been implicated in amyloid-beta (Aβ) formation [12] and phosphorylation of tau [13] as well, the neuropathological hallmarks of AD. The LPAs may contribute to amyloid pathology, which is supported by their role in enhancing Aβ production through upregulation β-secretase expression [12]. Moreover, as a bioactive component of oxidized LDL (OxLDL), LPAs affect the integrity of the blood-brain barrier [14] and are also involved in neuronal cell death [15, 16]. The mounting evidence for the role of LPA metabolites as a mediator in AD-related molecular process underline their importance in AD pathophysiology. Nevertheless, studies are lacking investigating the relationship between LPA metabolites with AD biomarkers of pathophysiology.

Encompassing a large group of related metabolites, LPA (1 or 2-acyl-sn-glycero-3-phosphate) metabolites comprises of an sn-glycerol-3 phosphate connected to a fatty acid [17–19]. Molecular species of LPAs differ based on their acyl chain length (C14 to C22) and degrees of saturation (C16:1, C18:1, C18:2, C20:3, C20:5, C20:4, C22:4, C22:6) [20]. For example, C18:1 denoting an acyl chain of 18 carbons with a double bond, while C18:2 denotes an acyl chain of 18 carbons with two double bonds [21, 22]. This structural diversity of LPAs also imparts them with differential biological activity [23–25]. Differential biological activity of LPAs can also be attributed to their G-coupled-protein-receptors (GCPR) ranging from LPA_1–5,_ which differ in their affinity and response to diverse LPA species [20, 25]. This structure-activity relationship of LPAs may be relevant to their role in AD pathophysiology [26]. A comprehensive study of LPA metabolites in AD-related pathology is lacking [14], and the interaction of apolipoprotein E (*APOE)* has not been studied. It is also not known whether LPAs play a role in the progression from mild cognitive impairment (MCI) to AD dementia.

Our study aims to delineate the role of various LPA species in AD during the prodromal phase of AD, i.e., MCI. We hypothesize that cerebrospinal fluid (CSF) and plasma levels of LPAs may be associated to markers of AD pathology, including to Aβ-42, phosphorylated tau (p-tau), and total tau (t-tau) in MCI patients, and this association may be modified by *APOE gene*. We further hypothesized that LPAs might contribute to MCI to AD dementia progression. As prior information on which LPA species may be relevant for the hypothesis, we assessed a series of structurally different LPA metabolites in CSF and plasma.

## Methods

### Study populations

The current study was performed in cohorts participating in the Alzheimer’s Disease Apolipoprotein Pathology for Treatment Elucidation and Development (ADAPTED) consortium including the Barcelona-based memory clinic Fundació ACE (142 CSF-plasma paired samples) and the Department of Geriatric Psychiatry at the Medical Faculty Mannheim, University of Heidelberg (40 CSF samples). Both participating studies are approved by the medical ethical committee of their respective institutes and informed consents were collected from all participants, which allow the use of phenotype and biomarker information for research purpose. From both participating cohorts, we selected MCI patients for which complete information was available on age at blood collection, sex, body mass index (BMI), and lipid-lowering medication use, as well as AD biomarkers in CSF (i.e., Aβ-42, p-tau, and total tau).

### Fundació ACE cohort

All the MCI patients from the Fundació ACE (ACE) cohort were recruited and assessed between 2016 to 2017 at the Memory Disorders Unit from Fundació ACE, Institut Català de Neurociènces Aplicades, Barcelona, Spain [27]. Each patient was assigned a diagnosis after consensus at a case conference attended by neurologists, neuropsychologists, and social workers. MCI patients fulfilled MCI Petersen’s diagnostic criteria [28, 29] including subjective memory complaints, decline from normal general cognition, preserved performance in activities of daily living, absence of dementia, and a measurable impairment in one or more cognitive functions, with or without deficit in other cognitive domains (amnestic MCI: single domain or amnestic MCI: multiple domain). At follow-up, dementia was defined according to the DSM-V criteria [30]. The underlying etiologies of the cognitive deficits within the dementia group were classified according to the following criteria: the 2011 National Institute of Aging-Alzheimer’s Association (NIA-AA) [31] for Alzheimer’s disease and the National Institute of Neurological Disorder and Stroke and Association Internationale pour la Recherche et l’Enseignement in Neurosciences criteria(NINDS-AIREN) [32] for vascular dementia, frontotemporal dementia [33], and Lewy body dementia [34].

Paired samples of CSF and plasma were collected from patients under fasted conditions. CSF was obtained by lumbar puncture following the established consensus recommendations [35]. Briefly, the lumbar puncture (LP) was performed by an experienced neurologist with the patients in a sitting position. After local anesthesia (1% mepivacaine) was injected subcutaneously, CSF was obtained by LP in the intervertebral space of L3-L4. The fluid was collected passively in two 10-ml polypropylene tubes (Sarstedt ref. 62610018). The first tube of CSF was analyzed for basic biochemistry (glucose, total proteins, proteinogram, and cell type and cell number). The second tube was centrifuged (2000×*g* 10 min at 4 °C), and the fluid was aliquoted into polypropylene tubes (Sarstedt ref. 72694007) and stored at − 80 °C until analysis. The time delay between CSF collection and storage was less than 2 h. On the same day as the AD biomarker analysis (Aβ-42, p-tau, and total tau), an aliquot was thawed at room temperature and vortexed for 5–10 s. CSF Aβ1–42, total tau, and p-tau levels were measured using commercially available enzyme-linked immunosorbent assays, namely Innotest Aβ1–42, Innotest hTAU Ag, and Innotest PHOSPHO-TAU (181P) (Innotest, Fujirebio Europe) [35, 36].

For *APOE* genotyping in the ACE cohort, genomic DNA was obtained from whole blood collected in BD Vacutainer tubes (K2-EDTA). DNA extraction was performed using DNA Chemagen technology (Perkin Elmer). Afterward, the *APOE* genotype was determined by TaqMan probes analysis in a system of Real-Time PCR QuantStudio3 (Thermofisher).

### Heidelberg/Mannheim memory clinic sample

Forty MCI patients were recruited and assessed between 2012 to 2016 at the Memory Clinic of the Central Institute of Mental Health (Mannheim, Germany). Neuropsychiatric or general medical causes of impaired cognition were excluded by detailed medical history, physical and neuropsychiatric examination, and standard serum laboratory assessment. Thus, all MCI patients met the MCI Petersen’s diagnostic criteria [28, 29] including subjective memory complaints, normal general cognition, only minimally impaired performance in instrumental activities of daily living, absence of dementia, and a measurable impairment in one or more cognitive domains. Cognitive impairment was defined as performance below 1.2 standard deviation in one or more cognitive domains in standard neuropsychological test battery [37] (test battery of the Consortium to Establish a Registry for Alzheimer Disease (CERAD) [38] plus the Wechsler memory scale – logical memory (WMS) immediate and delayed recall [39] and the trail making test A (TMT-A) and B (TMT-B) [40]. For biomarker assessments, lumbar puncture was performed to determine amyloid pathology in CSF following the NIA/AA criteria for the diagnosis of MCI due to AD [41]. The results of the clinical assessment for each patient were discussed at a case conference attended by geriatric psychiatrists and neuropsychologists. The diagnosis of MCI due to AD or prodromal AD [42] was assigned by consensus using all clinical and biomarker data (CSF Aβ-42, t-tau, and p-tau). Paired samples of CSF and plasma were collected from patients according to the established consensus recommendations [35]. Aliquots were stored in polypropylene tubes at − 80 °C. Aβ1–42, p-tau, and t-tau were performed in the Neurochemistry Laboratory at the Department of Neurology, University Medical School, Göttingen, using established protocols. P-tau levels in CSF were quantified with a commercially available ELISA kit [INNOTEST® PHOSPHO-TAU(181P), Innogenetics]. Aβ1–42 was detected with a commercially available ELISA kit [INNOTEST®β- AMYLOID (1–42) Innogenetics] for quantitative analysis.

*APOE* genotyping in Heidelberg/Mannheim memory clinic sample was performed on an Illumina GSA1.0 SharedCustom Content bead array according to the manufacturer’s instructions. GenomeStudio 2.0 software was used to determine *APOE* genotypes and results were exported in PLINK format.

### Metabolomics profiling

All CSF and plasma samples of both cohorts were profiled for the same set of metabolites using a UHPLC-MS/MS approach targeting signaling lipid mediators including LPAs, alkyl-lysophosphatidic acid (aLPAs), and cyclic-lysophosphatidic acids (cLPAs) ranging from C14 to C22 acyl chain length [43].

Samples were stored at − 80 °C, thawed at room temperature, and randomized prior to analysis. Quality control (QC) samples, consisting of a pool of all samples, and blanks were also analyzed to ensure the quality of the obtained data. For CSF samples, 350 μL of samples were evaporated to dryness, spiked with isotopically labeled internal standards and antioxidant (BHT:EDTA 1:1, 0.2 mg/mL), and reconstituted in two aliquots using a mixture of methanol to water (70:30, v/v). Plasma samples were first acidified through the addition of 0.2 M citric acid and 0.1 M disodium hydrogen phosphate buffer at pH 4.5. Metabolites were extracted using liquid-liquid extraction with a mixture of 1-butanol:ethyl acetate (1:1, v/v) prior to mixing, centrifugation, collection of the supernatant, evaporation, and reconstitution into two aliquots with a mixture of ice-cold methanol to water (70:30, v/v).

Samples were measured using a Shimadzu LC-30 AD system coupled to a LCMS-8050 Triple Quadrupole system (Shimadzu, Japan).

For both plasma and CSF samples, the first aliquot (high pH injection) was analyzed using a Kromasil EternityXT-1.8 C18 column, 2.1 × 50 mm, 1.8 μm (Akzo Nobel, Netherlands) with a mobile phase composed of (A) water with 5 mM ammonium acetate and 0.0625% ammonium hydroxide and (B) 80% acetonitrile with 20% isopropanol and 0.1% ammonium hydroxide. For both matrices, the second aliquot (low pH injection) was analyzed using an Acquity BEH C18 column, 2.1 × 50 mm, 1.7 μm (Waters) with a mobile phase composed of (A) water with 0.1% acetic acid, (B) 75% acetonitrile with 25% methanol and 0.1% acetic acid, and (C) 100% isopropanol. For both pH injections, polarity switching and dynamic multiple reaction monitoring (dMRM) mode were used for MS acquisition.

To perform the QC, metabolites showing a relative standard deviation (RSD) higher than 30% on corrected peak areas in QC samples were excluded. After QC correction, 19 and 17 LPAs in CSF and plasma, respectively, were used for further data analysis (Supplementary Table 1). Common metabolites detected in both CSF and plasma included LPAs (C14:0, C14:1, C14:2, C16:0, C18:0, C18:1, C18:2, C20:1, C20:3, C20:5, C22:4, C22:5) and three cyclic-LPAs (C16:0, C18:0, C18:1). Metabolites only detected in CSF samples included some LPAs (C20:4, C22:6, C22:5) and an alkyl-LPA C16:1. LPA C18:3 and two cyclic-LPAs (C18:2, C20:4) were detected only in plasma samples. The inverse rank transformation was used to normalize the distribution of metabolites in both cohorts.

### Statistical analysis

#### Association of LPAs with Aβ-42, p-tau, and t-tau

We performed linear regression analysis to test the association of Aβ-42, p-tau, and t-tau with the profiled metabolites in paired CSF and plasma samples from the ACE cohort and CSF samples from Heidelberg-Manheim memory clinic. Levels of Aβ-42, p-tau, and t-tau in CSF were used as the outcome variable in the regression model. Analyses were adjusted for age, sex, body mass index (BMI), and lipid-lowering medications. The inverse rank transformation was applied to normalize the distribution of both CSF AD biomarkers (Aβ-42, p-tau, and t-tau) and LPA metabolite levels in CSF and plasma. A meta-analysis of the regression analysis results of the two cohorts was performed using METAL software [44] using the inverse-variance fixed-effect model. Meta-analysis results of associations were also corrected for multiple testing separately for each AD biomarker using false discovery rate (*FDR*) by the Benjamini and Hochberg method [45] and findings with *FDR* < 0.05 were considered significant in the overall analysis. All analyses were performed in R (https://www.r-project.org/). To test whether conversion from LPA to another was relevant, we have tested all ratios between LPAs.

#### APOE-stratified regression analysis

To identify *APOE*-specific associations of metabolites with AD biomarkers, *APOE*-stratified analyses were performed in both participating cohorts based on three *APOE* strata including *APOE 44/34/24*, *APOE 33*, and *APOE 22/23*. In the stratified analyses, subjects with *APOE 24* genotype were pooled with patients having *APOE 44/34* genotypes based on their similar risk profiles, as reported in an earlier study [46]. *APOE*-stratified analyses results were reported as a combined meta-analysis of both datasets included in the current study (ACE cohort and Heidelberg/Manheim cohort). Due to the smaller number of *APOE 22/23* carriers in the two datasets, a combined regression analysis was performed, aggregating all *APOE 22/23* carriers from two cohorts while adjusting for cohort effects. Multiple testing correction was performed using the false-discovery rate (*FDR < 0.05*) based on Benjamin and Hochberg [45].

To assess the association of the *APOE* genotype with LPAs, we compared levels of LPAs in CSF of *APOE* ε4 (*APOE 44/34/24*) and *APOE* ε2 (22/23) carriers versus *APOE* ε33 carriers using regression analysis adjusting for the age, sex, BMI, and lipid-lowering medications. This regression analysis was conducted for each cohort and their combined meta-analysis.

#### MCI to AD dementia progression analysis

In the ACE cohort, follow-up information was available for 138 out of 142 MCI patients including 17 non-amnestic and 121 amnestic MCIs. A total of 43 MCI patients progressed into AD dementia (31%) during follow-up, while 95 MCI patients did not convert to AD dementia. The mean follow-up time in converters was 1.42 years (SD = 0.53) and 1.44 years (SD = 0.70) in non-converters. The rate of MCI to AD dementia progression in our sample is similar to other clinic-based studies [47]. We analyzed the association of LPAs with MCI to AD dementia progression using the cox proportional hazard model adjusting for age at blood collection, sex, BMI, and lipid-lowering medication use. In the ACE cohort, 11 MCI patients also progressed to other types of dementia including vascular dementia (*n* = 6), semantic dementia (*n* = 1), Parkinson dementia (*n* = 1), Lewy Body dementia (*n* = 2), and frontal temporal dementia (*n* = 1). We repeated the conversion analysis in the Heidelberg/Mannheim cohort. Among the 40 MCIs, 23 converted to AD dementia. The mean follow-up time in the Heidelberg/Mannheim cohort was 1.80 years (SD = 1.06). Three MCI patients also progressed to frontal temporal dementia in this sample.

#### Association of cognitive measures with LPA levels

We also assessed the association of cognitive measures, MMSE, and CDR with LPAs levels in CSF of both ACE and Heidelberg/Mannheim cohort. We used linear regression analysis adjusted for age, sex, BMI, and lipid-lowering medication. Results were meta-analyzed using METAL software [44] using the inverse-variance fixed-effect model and multiple testing was performed using false discovery rate (*FDR*) by the Benjamini and Hochberg method [45].

## Results

### General characteristics

The general characteristic of the ACE and Heidelberg/Mannheim cohorts are provided in Table 1. The patients of the ACE cohort of Barcelona are on average 3 years older (*P* = 0.042) than the Heidelberg/Mannheim cohort. The proportion of women is similar between the two cohorts (*P* = 0.747). The proportion of patients treated with lipid-lowering medication in the ACE cohort (44%) is 1.6 times (*P* = 0.055) higher compared to that in the Heidelberg/Mannheim series of patients. The levels of Aβ-42, p-tau, and t-tau in CSF between the two cohorts were not significantly different. In terms of basic cognitive measures, the Mini-Mental State Examination (MMSE) score (*P* = 2.67 × 10^−3^) and clinical dementia rating (CDR) score (*P* = 0.047) was significantly higher in Heidelberg/Mannheim cohort compared to ACE cohort.

**Table 1:** Population descriptive

	ACE cohort	Heidelberg/Mannheim cohort	***P*** value of difference
MCI patients (*N*)	142	40	
Metabolomics profiling tissue	CSF and plasma	CSF	
Age (SD) blood collection, years	71.94 (7.74)	68.85 (8.50)	0.042
Female (%)	74 (52%)	22 (55%)	0.747
Body mass index (SD)	26.46 (3.74)	25.85 (3.61)	0.353
Lipid-lowering medication user (%)	63 (44%)	11 (27%)	0.055
Amyloid-beta 42 in pg/mL (SD)	791.59 (337.36)	690.84 (397.13)	0.151
P-Tau in pg/mL (SD)	71.37 (37.30)	63.17 (29.96)	0.153
Total tau in pg/mL (SD)	478.82 (253.45)	380.95 (326.97)	0.124
MMSE	24.93 (4.07)	26.55 (2.51)	2.67 × 10^−3^
CDR	0.50 (0.06)	0.55 (0.15)	0.047
*APOE* genotype N (%)			
*APOE* 44/34/24	50 (35%)	18 (45%)	
*APOE* 33	81 (57%)	18 (45%)	
*APOE* 22/23	11 (8%)	4 (10%)	

*Abbreviations*: *MCI* mild cognitive impairment, *SD* standard deviation, *CSF* cerebrospinal fluid, *MMSE* the Mini-Mental State Examination, *CDR* clinical dementia rating, *APOE* apolipoprotein E gene

### Association of LPAs with CSF Aβ-42, p-tau, and total tau

Findings of the association of the metabolites with Aβ-42 levels in CSF are provided in Table 2. In a meta-analysis, eight LPAs including C18:1 (*β* = 0.281, *P* = 1.65 × 10^−3^), C16:1 (*β* = 0.242, *P* = 1.88 × 10^−3^), C16:0 (*β* = 0.234, *P* = 2 × 10^−3^), C22:6 (*β* = 0.231, *P* = 2.91 × 10^−3^), C14:0 (*β* = 0.223, *P* = 6.04 × 10^−3^), C22:4 (*β* = 0.191, *P* = 1.20 × 10^−2^), C20:4 (*β* = 0.203, *P* = 1.44 × 10^−2^), and isomer-LPA C22:5 (*β* = 0.183, *P* = 1.78 × 10^−2^) showed positive association with Aβ-42 levels in CSF. The effect estimates (*β*) of all associated LPAs were in the same direction in both cohorts.

**Table 2:** Association of cerebrospinal fluid (CSF) level of metabolites with Aβ-42 levels in CSF

	ACE cohort			Heidelberg/Mannheim cohort			Meta-analysis				
	***β***	SE	***P*** value	***β***	SE	***P*** value	***β***	SE	Direction	***P*** value	***FDR***
LPA C18:1	0.286	0.100	4.85 × 10^−3^	0.260	0.199	2.00 × 10^−1^	0.281	0.089	++	1.65 × 10^−3^	1.26 × 10^−2^
LPA C16:1	0.264	0.087	2.81 × 10^−3^	0.150	0.175	3.97 × 10^−1^	0.242	0.078	++	1.88 × 10^−3^	1.26 × 10^−2^
LPA C16:0	0.249	0.081	2.61 × 10^−3^	0.134	0.209	5.27 × 10^−1^	0.234	0.076	++	2.00 × 10^−3^	1.26 × 10^−2^
LPA C22:6	0.199	0.086	2.19 × 10^−2^	0.372	0.181	4.76 × 10^−2^	0.231	0.078	++	2.91 × 10^−3^	1.38 × 10^−2^
LPA C14:0	0.220	0.092	1.76 × 10^−2^	0.232	0.175	1.92 × 10^−1^	0.223	0.081	++	6.04 × 10^−3^	2.29 × 10^−2^
LPA C22:4	0.150	0.084	7.59 × 10^−2^	0.393	0.184	4.02 × 10^−2^	0.191	0.076	++	1.20 × 10^−2^	3.79 × 10^−2^
LPA C20:4	0.211	0.096	2.91 × 10^−2^	0.178	0.165	2.89 × 10^−1^	0.203	0.083	++	1.44 × 10^−2^	3.90 × 10^−2^
Isomer-LPA C22:5	0.208	0.086	1.61 × 10^−2^	0.071	0.177	6.90 × 10^−1^	0.183	0.077	++	1.78 × 10^−2^	4.23 × 10^−2^
LPA C18:0	0.172	0.090	5.65 × 10^−2^	0.211	0.176	2.39 × 10^−1^	0.180	0.080	++	2.39 × 10^−2^	5.05 × 10^−2^
LPA C18:2	0.189	0.083	2.51 × 10^−2^	0.028	0.170	8.69 × 10^−1^	0.158	0.075	++	3.50 × 10^−2^	6.65 × 10^−2^
cLPA C18:1	0.170	0.093	7.03 × 10^−2^	0.055	0.186	7.68 × 10^−1^	0.147	0.083	++	7.77 × 10^−2^	1.34 × 10^−1^
LPA C22:5	0.112	0.088	2.04 × 10^−1^	0.230	0.179	2.09 × 10^−1^	0.135	0.079	++	8.70 × 10^−2^	1.35 × 10^−1^
LPA C20:1	0.164	0.092	7.59 × 10^−2^	0.038	0.175	8.29 × 10^−1^	0.137	0.081	++	9.21 × 10^−2^	1.35 × 10^−1^
cLPA C16:0	0.199	0.105	5.91 × 10^−2^	− 0.046	0.187	8.06 × 10^−1^	0.141	0.091	+−	1.23 × 10^−1^	1.67 × 10^−1^
aLPA C18:1	0.335	0.127	9.31 × 10^−3^	− 0.225	0.174	2.04 × 10^−1^	0.140	0.103	+−	1.72 × 10^−1^	2.17 × 10^−1^
cLPA C18:0	0.085	0.091	3.54 × 10^−1^	0.036	0.193	8.55 × 10^−1^	0.076	0.082	++	3.58 × 10^−1^	4.25 × 10^−1^
aLPA C16:1	0.199	0.132	1.35 × 10^−1^	− 0.106	0.166	5.30 × 10^−1^	0.081	0.103	+−	4.33 × 10^−1^	4.84 × 10^−1^
LPA C20:3	0.124	0.103	2.29 × 10^−1^	− 0.104	0.169	5.41 × 10^−1^	0.062	0.088	+−	4.77 × 10^−1^	5.04 × 10^−1^
LPA C20:5	− 0.114	0.090	2.08 × 10^−1^	0.253	0.185	1.80 × 10^−1^	− 0.044	0.081	−+	5.88 × 10^−1^	5.88 × 10^−1^

Direction column indicates the direction of regression coefficient of association in the ACE and Heidelberg/Mannheim cohort respectively

*Abbreviations*: *LPA* lysophosphatidic acid, *cLPA* cyclic lysophosphatidic acid, *aLPA* alkyl-Lysophosphatidic acid, *SE* standard error, *FDR* false discovery rate

Six LPAs, C20:1 (*β* = 0.347, *P* = 7.11 × 10^−6^), isomer-LPA C22:5 (*β* = 0.328, *P* = 8.68 × 10^−6^), C22:6 (*β* = 0.270, *P* = 4.03 × 10^−4^), C16:0 (*β* = 0.230, *P* = 2.26 × 10^−3^), C16:1 (*β* = 0.206, *P* = 8.23 × 10^−3^), and C22:4 (*β* = 0.186, *P* = 1.39 × 10^−2^) showed significant association (*FDR* < 0.05) with p-tau levels in CSF. In terms of the direction of effects, the regression coefficients (*β*) were very similar across two cohorts (Table 3).

**Table 3:** Association of cerebrospinal fluid (CSF) level of metabolites with p-tau levels in CSF

	ACE cohort			Heidelberg/Mannheim cohort			Meta-analysis				
	***β***	SE	***P*** value	***β***	SE	***P*** value	***β***	SE	Direction	***P*** value	***FDR***
LPA C20:1	0.350	0.086	8.16 × 10^−5^	0.333	0.174	6.42 × 10^−2^	0.347	0.077	++	7.11 × 10^−6^	8.25 × 10^−5^
Isomer-LPA C22:5	0.302	0.082	3.15 × 10^−4^	0.438	0.170	1.46 × 10^−2^	0.328	0.074	++	8.68 × 10^−6^	8.25 × 10^−5^
LPA C22:6	0.279	0.083	9.39 × 10^−4^	0.214	0.198	2.88 × 10^−1^	0.270	0.076	++	4.03 × 10^−4^	2.55 × 10^−3^
LPA C16:0	0.218	0.080	7.67 × 10^−3^	0.316	0.214	1.48 × 10^−1^	0.230	0.075	++	2.26 × 10^−3^	1.07 × 10^−2^
LPA C16:1	0.226	0.086	9.49 × 10^−3^	0.112	0.182	5.43 × 10^−1^	0.206	0.078	++	8.23 × 10^−3^	3.13 × 10^−2^
LPA C22:4	0.168	0.082	4.17 × 10^−2^	0.287	0.196	1.53 × 10^−1^	0.186	0.076	++	1.39 × 10^−2^	4.40 × 10^−2^
LPA C18:1	0.217	0.099	3.08 × 10^−2^	0.171	0.212	4.25 × 10^−1^	0.209	0.090	++	2.04 × 10^−2^	5.54 × 10^−2^
aLPA C18:1	0.110	0.128	3.91 × 10^−1^	0.425	0.169	1.69 × 10^−2^	0.224	0.102	++	2.79 × 10^−2^	6.62 × 10^−2^
cLPA C18:0	0.159	0.089	7.53 × 10^−2^	0.243	0.197	2.26 × 10^−1^	0.173	0.081	++	3.23 × 10^−2^	6.82 × 10^−2^
LPA C20:3	0.072	0.101	4.79 × 10^−1^	0.380	0.163	2.62 × 10^−2^	0.157	0.086	++	6.77 × 10^−2^	1.29 × 10^−1^
cLPA C18:1	0.115	0.092	2.14 × 10^−1^	0.265	0.189	1.69 × 10^−1^	0.144	0.083	++	8.25 × 10^−2^	1.42 × 10^−1^
LPA C14:0	0.129	0.091	1.60 × 10^−1^	0.088	0.187	6.42 × 10^−1^	0.121	0.082	++	1.40 × 10–^1^	2.12 × 10^−1^
LPA C22:5	0.065	0.087	4.56 × 10^−1^	0.326	0.184	8.51 × 10^−2^	0.112	0.078	++	1.52 × 10^−1^	2.12 × 10^−1^
aLPA C16:1	0.061	0.131	6.42 × 10^−1^	0.287	0.168	9.65 × 10^−2^	0.146	0.103	++	1.56 × 10^−1^	2.12 × 10^−1^
LPA C18:0	0.096	0.089	2.82 × 10^−1^	0.077	0.188	6.86 × 10^−1^	0.092	0.080	++	2.50 × 10^−1^	3.16 × 10^−1^
LPA C18:2	− 0.094	0.083	2.60 × 10^−1^	−0.024	0.175	8.90 × 10^−1^	− 0.081	0.075	–	2.79 × 10^−1^	3.30 × 10^−1^
cLPA C16:0	0.102	0.104	3.28 × 10^−1^	0.075	0.196	7.04 × 10^−1^	0.096	0.092	++	2.95 × 10^−1^	3.30 × 10^−1^
LPA C20:4	0.077	0.095	4.20 × 10^−1^	0.081	0.176	6.49 × 10^−1^	0.078	0.084	++	3.53 × 10^−1^	3.72 × 10^−1^
LPA C20:5	− 0.028	0.088	7.54 × 10^−1^	0.114	0.193	5.59 × 10^−1^	− 0.003	0.081	−+	9.68 × 10^−1^	9.68 × 10^−1^

Direction column indicates the direction of regression coefficient of association in the ACE and Heidelberg/Mannheim cohort respectively

*Abbreviations*: *LPA* lysophosphatidic acid, *cLPA* cyclic lysophosphatidic acid, *aLPA* alkyl-Lysophosphatidic acid, *SE* standard error, *FDR* false discovery rate

Findings for t-tau levels in CSF were very similar as those for p-tau levels (Table 4), with five LPAs including C20:1, isomer-LPA C22:5, C22:6, C16:0, C16:1, C22:4, C18:1, and alkyl-LPA C18:1 showing significant positive association (*FDR* < 0.05) with total tau levels in CSF. Among the identified metabolites, LPA C18:1 and alkyl-LPA C18:1 showed association with only total tau but not with p-tau levels.

**Table 4:** Association of cerebrospinal fluid (CSF) level of metabolites with t-tau levels in CSF

	ACE cohort			Heidelberg/Mannheim cohort			Meta-analysis				
	***β***	SE	***P*** value	***β***	SE	***P*** value	***β***	SE	Direction	***P*** value	***FDR***
LPA C20:1	0.318	0.085	2.89 × 10^−4^	0.391	0.181	3.76 × 10^−2^	0.331	0.077	++	1.79 × 10^−5^	3.40 × 10^−4^
Isomer-LPA C22:5	0.271	0.081	1.05 × 10^−3^	0.390	0.184	4.14 × 10^−2^	0.290	0.074	++	8.88 × 10^−5^	8.07 × 10^−4^
LPA C22:6	0.268	0.081	1.22 × 10^−3^	0.409	0.200	4.84 × 10^−2^	0.288	0.075	++	1.27 × 10^−4^	8.07 × 10^−4^
LPA C16:0	0.227	0.079	4.42 × 10^−3^	0.461	0.218	4.18 × 10^−2^	0.254	0.074	++	5.80 × 10^−4^	2.75 × 10^−3^
LPA C16:1	0.236	0.084	5.67 × 10^−3^	0.280	0.187	1.44 × 10^−1^	0.244	0.077	++	1.48 × 10^−3^	5.63 × 10^−3^
LPA C22:4	0.185	0.080	2.21 × 10^−2^	0.361	0.203	8.51 × 10^−2^	0.209	0.074	++	5.05 × 10^−3^	1.60 × 10^−2^
LPA C18:1	0.230	0.097	1.93 × 10^−2^	0.190	0.223	3.99 × 10^−1^	0.224	0.089	++	1.20 × 10^−2^	3.26 × 10^−2^
aLPA C18:1	0.125	0.125	3.18 × 10^−1^	0.488	0.175	8.52 × 10^−3^	0.248	0.102	++	1.46 × 10^−2^	3.48 × 10^−2^
LPA C20:3	0.070	0.099	4.83 × 10^−1^	0.504	0.163	4.06 × 10^−3^	0.187	0.085	++	2.78 × 10^−2^	5.86 × 10^−2^
LPA C14:0	0.156	0.089	8.13 × 10^−2^	0.196	0.194	3.19 × 10^−1^	0.163	0.081	++	4.36 × 10^−2^	7.58 × 10^−2^
cLPA C18:0	0.166	0.087	5.88 × 10^−2^	0.140	0.210	5.11 × 10^−1^	0.162	0.080	++	4.39 × 10^−2^	7.58 × 10^−2^
aLPA C16:1	0.093	0.128	4.70 × 10^−1^	0.377	0.172	3.54 × 10^−2^	0.194	0.103	++	5.89 × 10^−2^	9.32 × 10^−2^
cLPA C18:1	0.110	0.090	2.26 × 10^−1^	0.202	0.201	3.21 × 10^−1^	0.126	0.082	++	1.28 × 10^−1^	1.87 × 10^−1^
LPA C20:4	0.084	0.094	3.68 × 10^−1^	0.245	0.180	1.83 × 10^−1^	0.119	0.083	++	1.54 × 10^−1^	2.08 × 10^−1^
LPA C18:0	0.112	0.087	2.01 × 10^−1^	0.050	0.198	8.03 × 10^−1^	0.102	0.080	++	2.01 × 10^−1^	2.55 × 10^−1^
LPA C22:5	0.026	0.085	7.59 × 10^−1^	0.407	0.189	3.88 × 10^−2^	0.090	0.078	++	2.46 × 10^−1^	2.92 × 10^−1^
cLPA C16:0	0.110	0.102	2.83 × 10^−1^	0.021	0.207	9.18 × 10^−1^	0.092	0.091	++	3.11 × 10^−1^	3.48 × 10^−1^
LPA C18:2	− 0.041	0.082	6.19 × 10^−1^	0.106	0.183	5.66 × 10^−1^	− 0.016	0.075	−+	8.27 × 10^−1^	8.60 × 10^−1^
LPA C20:5	− 0.007	0.087	9.39 × 10^−1^	0.128	0.203	5.34 × 10^−1^	0.014	0.080	−+	8.60 × 10^−1^	8.60 × 10^−1^

Direction column indicates the direction of regression coefficient of association in the ACE and Heidelberg/Mannheim cohort respectively

*Abbreviations*: *LPA* lysophosphatidic acid, *cLPA* cyclic lysophosphatidic acid, *aLPA* alkyl-Lysophosphatidic acid, *SE* standard error, *FDR* false discovery rate

**Fig. 1:**
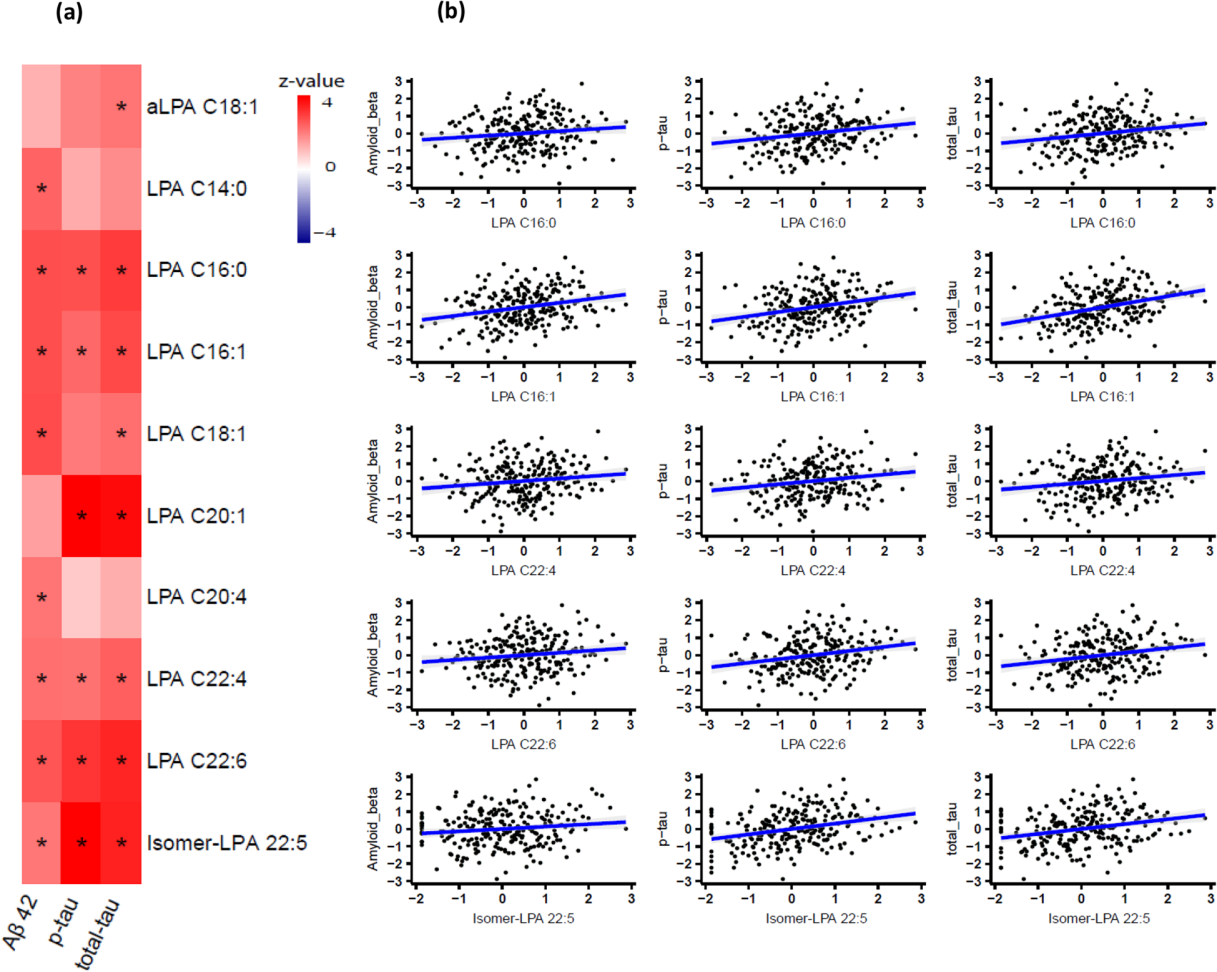
**a** Heatmap of overall meta-analysis of regression analysis results of metabolites with Aβ-42, p-tau, and t-tau levels in cerebrospinal fluid (CSF). Star indicates significant association with false discovery rate < 0.05. **b** Scatter plots of correlation between LPAs (C16:0. C16:1, C22:4, C22:6, isomer-LPA C22) with Aβ-42, p-tau, and t-tau levels in CSF

Figure 1a shows a heatmap comparing Aβ-42, p-tau, and t-tau in CSF. The pattern of association is very similar for amyloid and tau biomarkers except for two LPAs (C14:0, C20:4) showing association to only Aβ-42 and an alkyl-LPA C18:1 showing a unique relation to total tau levels in CSF. Correlation plots are provided in Fig. 1 for five LPAs (C16:0. C16:1, C22:4, C22:6, isomer-LPA C22:5), which showed significant association with all three AD biomarkers (Aβ-42, p-tau, and t-tau). The analysis of the ratios did not yield any significant findings.

In the regression analysis of LPAs in plasma (Supplementary Table 2), no significant association was found with Aβ-42, p-tau, or with t-tau in ACE cohort. Correlation analysis between CSF and plasma levels of LPAs for which paired samples were available (Supplementary Fig. 1) shows that levels of 8 LPAs were significantly correlated between CSF and plasma: cyclic-LPA C16:0 (*R* = − 0.28, *P* = 9.4 × 10^−4^), cyclic-LPA C18:1 (*R* = − 0.18, *P* = 0.029), LPA C18:1 (*R* = 0.44, *P* = 4.5 × 10^−8^), LPA C20:1 (*R* = − 0.23, *P* = 6.2 × 10^−3^), and LPA C20:5 (*R* = 0.4, *P* = 1.2 × 10^−6^).

### The role of *APOE* in the association between LPA levels and CSF AD biomarkers

**Fig. 2:**
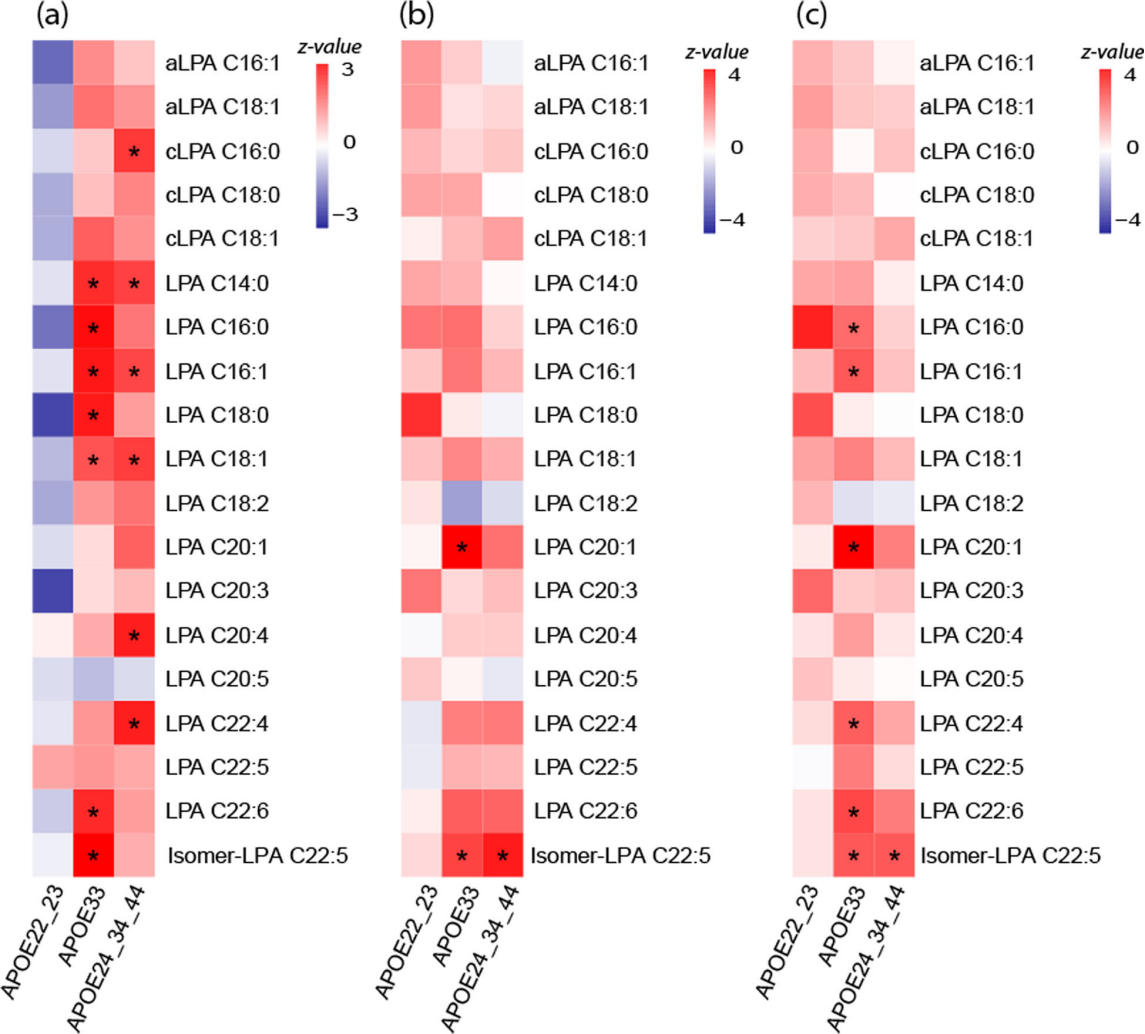
Heatmap of *APOE*-stratified meta-analysis of regression analysis results of metabolites with Aβ-42 (**a**), p-tau (**b**), and t-tau (**c**) levels in cerebrospinal fluid. Star indicates significant association with false discovery rate < 0.05 in each stratum

The results of meta-analysis of association results of *APOE*-stratified analyses are provided in Fig. 2. *APOE*-stratified analysis of Aβ-42 (Fig. 2a) showed that LPAs species which showed significant association in the overall meta-analysis (C14:0, C16:0, C16:1, C18:1, C20:4, C22:4, C22:6, isomer-LPA C22:5) were restricted to *APOE* ε33 (LPA C16:0, C18:0, C22:6, isomer-LPA C22:5) and *APOE* ε4 (LPA C20:4, C22:4) carriers while in *APOE* 22/23 carriers, the association was not significant and in the opposite direction. Based on statistical significance, we observed a few unique associations in the *APOE4* stratum involving a cyclic-LPA C16:0 and in the *APOE33* stratum LPAs C18:0, which did not show significant association in overall regression analysis. Although the level of statistical significance differed between *APOE* ε33 and *APOE* ε4 carriers, the direction of association was always similar.

For p-tau and t-tau levels, significant association was observed with isomer-LPA C22:5 in *APOE* 33 and *APOE* ε4 carriers (Fig. 2b, c). Further significant association was observed between LPA C20:1 and p-tau and three LPAs (C16:0, C16:1, C22:4) and t-tau only in *APOE* 33 carriers.

As *APOE* appeared to modify the association between various metabolites and CSF biomarkers, we tested whether the *APOE* genotype is associated with levels of the metabolites associated with biomarkers in the overall and *APOE*-stratified analyses (see Supplementary Table 3 and 4). We did not observe significant differences between *APOE* ε4 versus *APOE* ε33 carriers and *APOE* ε2 versus *APOE 33* carriers in the combined meta-analysis of the two datasets. Although cLPA C18:1 showed suggestive association with *APOE* ε2 in the combined meta-analysis (*β* = − 0.636, *P* = 4.66 × 10^−3^, *FDR* = 8.85 × 10^−2^) but did not pass multiple testing.

### MCI to AD dementia progression

In the ACE cohort, LPA C16:1 (*β* = − 0.472, *P* = 3.25 × 10^−3^, *FDR* = 4.41 × 10^−2^) and LPA C16:0 (*β* = − 0.412, *P* = 4.64 × 10^−3^, *FDR* = 4.41 × 10^−2^) were significantly associated with progression from MCI to AD dementia (Supplementary Table 5). LPA C16:1 and C16:0 levels in CSF are correlated with each other (Supplementary Fig. 2), which is also evident from their similar regression coefficient in the progression analysis. As a sensitivity analysis, we also performed cox proportional hazard analysis, additionally correcting for *APOE* (Supplementary Table 6) and Aβ-42 levels in CSF (Supplementary Table 7) to assess the role of LPAs in MCI to AD progression. *APOE* did not affect the association of LPA C16:0 and C16:1 to MCI to AD dementia progression while the association was lost upon adjusting for Aβ-42 levels. In plasma, LPA C20:1 (*β* = 0.599, *P* = 1.84 × 10^−2^) showed evidence of association to MCI to AD dementia progression but significance was lost upon adjusting for multiple testing (Supplementary Table 8). In the Heidelberg/Mannheim sample (Supplementary Table 9), we did not replicate our findings from ACE cohort, i.e., LPA C16:1 (*β* = 0.457, *P* = 8.91 × 10^−2^) and LPA C16:0 (*β* = 0.431, *P* = 1.96 × 10^−1^).

### Association of cognitive measures with LPA levels

We did not find significant association of LPA levels in CSF with MMSE and CDR score in the combined meta-analysis (Supplementary Tables 10 and 11). In individual cohorts, only alkyl-LPA C16:1 showed evidence of association with MMSE (*β* = 0.253, *P* = 4 × 10^−2^) in ACE cohort and with CDR score (*β* = 0.254, *P* = 6.01 × 10^−3^) in Heidelberg/Mannheim sample.

## Discussion

Meta-analysis of the data of two independent cohorts showed a significant association of eight LPAs to Aβ-42, six LPAs to p-tau, and eight LPAs to t-tau levels in CSF. In the *APOE*-stratified meta-analysis, one cyclic LPA C16:0 and two LPAs (C20:4, C22:4) associate significantly with Aβ-42 levels in *APOE* ε4 carriers only. The association of LPAs with p-tau and total tau were confined to *APOE* ε33 carriers except for isomer-LPA C22:5, which showed association in both *APOE33* and *APOE4* strata. LPA C16:0 and C16:1 were associated with the progression of MCI to AD in the ACE cohort but the association was no longer significant after adjusting for Aβ-42 in the model.

The positive association between various LPAs and Aβ-42 is in line with the findings of an earlier study, suggesting that LPAs play a role in Aβ production by upregulation of β-secretase (BACE1) [12], a key enzyme involved in the cleavage of amyloid precursor protein (*APP*). Our study pinpoints a key role of LPA C18:1, C16:1, C16:0, C22:6, C14:0, C22:4, C20:4, and isomer-LPA C22:5 in CSF amyloid levels, detailing which specific LPAs are relevant. The association of LPA to CSF amyloid sheds new light on the role of (signaling) lipids in AD pathogenies. LPAs are a bioactive component of oxLDL, which show a positive correlation with CSF levels of Aβ [48]. Moreover, traumatic brain injury (TBI) patients also exhibit increased CSF levels of LPAs [49]. Because amyloid pathology is observed in nearly 30% of TBI patients with unknown mechanisms [50], our observed positive association of amyloid pathology and LPA may suggest the role of LPAs as a mediator in the aggregation of amyloid pathology, which needs further investigation. On the other hand, Aβ may increase oxidative stress and inflammation, which results in the production of LPAs. There is a need for functional studies to ascertain whether the positive correlation between amyloid and LPA is a cause or consequence of pathological process.

In our study, CSF levels of LPAs (C16:0 and C16:1) were significantly associated with MCI to AD dementia progression in the ACE cohort. Similar to the inverse relation of CSF Aβ-42 levels in MCI to AD dementia progression, decreased levels of the LPAs are associated with MCI to AD dementia progression [51]. Loss of association of LPAs with MCI to AD dementia progression when accounting for Aβ-42 levels suggests that Aβ-42 mediates the observed association of LPA C16:0 and C16:1 in conversion. This is of note that LPA 16:0 and 16:1 did not show significant association with MCI to AD progression in the smaller Heidelberg/Mannheim sample. However, LPA 16:0 and 16:1 also did not show significant association with Aβ-42 levels in this small cohort, making findings difficult to interpret. The fact that the association LPA C16:0 and C16:1 to conversion loses its significance when adjusting for Aβ-42 levels suggests the LPA C16:0 and C16:1 are likely preceding the changes in Aβ-42 that predict conversion to AD.

In the *APOE*-stratified analysis, LPA C16:1 showed a positive association to Aβ-42 levels in both *APOE* ε4 and *APOE* ε33 carriers, whereas LPA C16:0 showed significant positive association in only *APOE* ε3 stratum. No effect of the ratio of these two correlated LPAs was seen. The findings imply that the association of these LPAs to Aβ-42 levels may be modified by *APOE* genotype of the person. To our knowledge, this is the first study that shows the *APOE* interacts with LPA in humans. Interestingly, all LPA showed a negative association with Aβ-42 in *APOE* ε2 carriers, i.e., in the opposite direction compared to *APOE* ε33 and *APOE* ε4 carrier. Since *APOE* ε2 carriers are protected from AD and have delayed onset of AD [46], LPA modification may be relevant in *APOE* ε33 and *APOE* ε4 carriers. We did not observe an association of the interacting LPAs with *APOE* genotypes, which may be due to a limited sample size in these analyses.

Except for LPA C16:0, we found that the majority of unsaturated LPAs (C16:1, C20:1, isomer-LPA C22:5, C22:6, and C22:4) showed significant association to both p-tau and total tau levels in CSF. Due to differential activation of LPA receptors by diverse LPA metabolite species [23], association of unsaturated LPAs with AD biomarkers of pathophysiology can also be explained by their affinity for LPA_3_ receptors [52] which are also expressed in hippocampus, frontal cortex, and amygdala [53]. Moreover, both saturated and unsaturated LPAs are reported to influence Ca^2+^ signaling through LPA2 receptors [52], which may also suggest their involvement in the dysregulation of Ca^2+^ signaling in AD. Earlier studies have shown that LPAs acts as mediators to maintain the intracellular Ca^2+^ levels in both astrocytes [54] and microglial cells [5, 55]. One of these LPAs (LPA C20:1) only showed significant association with p-tau and total tau levels but not with Aβ-42. These tau specific associations may be explained by the fact that LPAs are involved in the upregulation of glycogen synthase kinase-3 (GSK-3), an enzyme involved in phosphorylation of tau and thus may influence levels of p-tau in CSF [13, 56]. The association of LPA C20:1 to only tau pathology may also indicate the specificity of association of LPA species of different acyl chains to different AD pathophysiological mechanisms. In the *APOE*-stratified meta-analysis, all the observed associations were largely confined to *APOE* ε33 carriers except for isomer-LPA C22:5. This observation is in line with the studies which demonstrated that *APOE* ε4 may influence amyloid pathology in the brain rather than tau aggregation [57–59].

We did not observe association of LPA levels in plasma with Aβ-42, p-tau, and total-tau in CSF nor did we find association with MCI to AD dementia progression. It is interesting that we observed a significant correlation between CSF and plasma measurements of various LPA molecular species. A negative correlation was observed for cyclic-LPA C16:0, cyclic-LPA C18:1, and LPA C20:1. Of these, the association was strongest and most convincing for LPA C20:1 in terms of *R* (− 0.23) and *p* value 6.2 × 10^−3^. The positive correlations are more convincing in particular for LPA C18:1 (*R* = 0.44 and *P* = 4.5 × 10^−8^) and C20:5(*R* = 0.40 and *P* = 1.2 × 10^−6^) and LPA 22:4 (*R* = 0.27 and *P* = 1.2 × 10^−3^) (Supplementary Fig. 1). LPAs found associated with Aβ, p-tau and t-tau in CSF were not correlated with their counterparts in plasma, which indicates that the LPA role in relation to AD pathology is primarily cerebral and not in the circulation.

Our study provides a comprehensive overview of association of various LPA species including alkyl-LPAs and cyclic-LPAs to biomarkers of AD during the prodromal phase. The inclusion of two independent cohorts is a major strength of our investigation, allowing us to check consistency of effect across cohorts. Moreover, we have also assessed the role of LPAs longitudinally for MCI to AD dementia progression in the ACE cohort.

### Limitations

The short follow-up time for MCI patients in the progression study is a limitation of our study and asks for replication in a study with longer follow-up. The small sample size in MCI to AD progression analysis is another major limitation of our study. In the absence of any data on the association between LPA and AD biomarkers in CSF, we did not perform a power calculation a priori, which limits the clinical and predictive implications of the discovery analysis. Future large sample sizes in follow-up studies will also provide more power to perform *APOE*-stratified analysis.

## Conclusions

Overall findings from our study suggest that various LPAs based on acyl chain length and saturation level are associated with Aβ-42, p-tau, and total tau levels. Our study suggests the role of LPAs in the pathophysiology of AD. Future studies are needed to determine whether LPA metabolites triggers various biological pathways leading to increase in biomarkers of AD pathophysiology or are produced as a downstream effect of AD pathology. We further find that *APOE* may influence the association between LPAs and Aβ-42.

## Supplementary information

**Additional file 1: Table S1.** List of detected lysophosphatidic acids in cerebrospinal fluid and plasma. **Table S2.** Association of metabolites in plasma association with amyloid-beta 42, P-Tau and total tau. **Table S3.** Association of metabolites in cerebrospinal fluid with *APOE* 22/23 versus *APOE* 33. **Table S4.** Association of metabolites in cerebrospinal fluid with *APOE* 44/34/24 vs *APOE* 33. **Table S5.** Association of metabolites measured in CSF with MCI to AD conversion in ACE cohort. **Table S6.** Association of metabolites measured in CSF with MCI to AD conversion adjusting for *APOE* in ACE cohort. **Table S7.** Association of metabolites measured in CSF with MCI to AD conversion adjusted for amyloid beta 42 levels in ACE cohort. **Table S8.** Association of metabolites measured in plasma with MCI to AD conversion in ACE cohort. **Table S9.** Association of metabolites measured in CSF with MCI to AD conversion in Heidelberg/Mannheim sample. **Table S10.** Association of the Mini-Mental State Examination (MMSE) with LPAs in CSF. **Table S11.** Association of the clinical dementia score (CDR) with LPAs in CSF. **Figure S1.** Correlation of metabolite levels between plasma and CSF. **Figure S2.** Correlation matrix of CSF LPA metabolites.

## Data Availability

Data can only be available upon request for only research purposes. The availability of data will be possible in compliance with EU-GDPR rules. Data access requests can be made directly to the corresponding authors.

## Abbreviations

AD: Alzheimer’s disease
LPAs: Lysophosphatidic acids
*APOE*: Apolipoprotein E
MCI: Mild cognitive impairment
CSF: Cerebrospinal fluid
p-tau: Phosphorylated tau (p-tau)
t-tau: Total tau
Aβ: Amyloid-beta
ADAPTED: The Alzheimer’s Disease Apolipoprotein Pathology for Treatment Elucidation and Development consortium
BMI: Body mass index
ACE: The Fundació ACE cohort
FDR: False discovery rate
