# Supplementary material for: Association of lysophosphatidic acids with cerebrospinal fluid biomarkers and progression to Alzheimer’s disease
Source: Alzheimers Res Ther. 2020 Oct 2;12:124. doi: 10.1186/s13195-020-00680-9 (PMC7532619; doi:10.1186/s13195-020-00680-9)

| **Abbreviations** | **Name** | **Acyle chain length** | **Number of Double bonds** | **CSF** | **Plasma** | **Lipid maps ID** |
| --- | --- | --- | --- | --- | --- | --- |
| aLPA C16:1 | alkyl-Lysophosphatidic acid C16:1 | 16 | 1 | Yes | No |  |
| aLPA C18:1 | alkyl-Lysophosphatidic acid C18:1 | 18 | 1 | Yes | No |  |
| cLPA C16:0 | cyclic-Lysophosphatidic acid C16:0 | 16 | saturated | Yes | Yes | LMGP00000057 |
| cLPA C18:0 | cyclic-Lysophosphatidic acid C18:0 | 18 | saturated | Yes | Yes | LMGP00000055 |
| cLPA C18:1 | cyclic-Lysophosphatidic acid C18:1 | 18 | 1 | Yes | Yes | LMGP00000056 |
| cLPA C18:2 | cyclic-Lysophosphatidic acid C18:2 | 18 | 2 | No | Yes |  |
| cLPA C20:4 | cyclic-Lysophosphatidic acid C20:4 | 20 | 4 | No | Yes |  |
| LPA C14:0 | Lysophosphatidic acid C14:0 | 14 | saturated | Yes | Yes | LMGP10050007 |
| LPA C16:0 | Lysophosphatidic acid C16:0 | 16 | saturated | Yes | Yes | LMGP10050006 |
| LPA C16:1 | Lysophosphatidic acid C16:1 | 16 | 1 | Yes | Yes | LMGP10050016 |
| LPA C18:0 | Lysophosphatidic acid C18:0 | 18 | saturated | Yes | Yes | LMGP10050005 |
| LPA C18:1 | Lysophosphatidic acid C18:1 | 18 | 1 | Yes | Yes | LMGP10050008 |
| LPA C18:2 | Lysophosphatidic acidC18:2 | 18 | 2 | Yes | Yes | LMGP10050017 |
| LPA C18:3 | Lysophosphatidic acidC18:3 | 18 | 3 | No | Yes | LMGP10050023 |
| LPA C20:1 | Lysophosphatidic acid C20:1 | 20 | 1 | Yes | Yes | LMGP10050026 |
| LPA C20:3 | Lysophosphatidic acid C20:3 | 20 | 3 | Yes | Yes | LMGP10050028 |
| LPA C20:4 | Lysophosphatidic acid C20:4 | 20 | 4 | Yes | No | LMGP10050013 |
| LPA C20:5 | Lysophosphatidic acid C20:5 | 20 | 5 | Yes | Yes | LMGP10050033 |
| LPA C22:4 | Lysophosphatidic acidC22:4 | 22 | 4 | Yes | Yes | LMGP10050020 |
| LPA C22:5 | Lysophosphatidic acid C22:5 | 22 | 5 | Yes | No |  |
| LPA C22:6 | Lysophosphatidic acid C22:6 | 22 | 6 | Yes | No | LMGP10050019 |
| Isomer-LPA C22:5 | isomer-Lysophosphatidic acid C22:5 | 22 | 5 | Yes | Yes |  |

**Supplementary Table 1: List of detected lysophosphatidic acids in cerebrospinal fluid and plasma**

Abbreviations: CSF, cerebrospinal fluid

**Supplementary Table 2: Association of metabolites in plasma association with amyloid-beta 42, P-Tau and total tau**

|  | **Aβ42** | | | | **p-tau** | | | **Total tau** | | | | | |
| --- | --- | --- | --- | --- | --- | --- | --- | --- | --- | --- | --- | --- | --- |
|  | **β** | **SE** | ***P-value*** | ***FDR*** | **β** | **SE** | ***P-value*** | ***FDR*** | **β** | | **SE** | ***P-value*** | ***FDR*** |
| LPA C20:3 | 0.179 | 0.141 | 2.04E-01 | 8.89E-01 | -0.179 | 0.138 | 1.98E-01 | 8.12E-01 | | -0.169 | 0.135 | 2.14E-01 | 9.81E-01 |
| Isomer-LPA C22:5 | -0.105 | 0.090 | 2.44E-01 | 8.89E-01 | 0.009 | 0.089 | 9.23E-01 | 9.94E-01 | | 0.013 | 0.087 | 8.84E-01 | 9.81E-01 |
| cLPA C16:0 | -0.144 | 0.125 | 2.54E-01 | 8.89E-01 | 0.198 | 0.122 | 1.09E-01 | 8.12E-01 | | 0.187 | 0.120 | 1.22E-01 | 9.81E-01 |
| LPA C20:1 | -0.141 | 0.136 | 3.03E-01 | 8.89E-01 | 0.236 | 0.133 | 7.78E-02 | 8.12E-01 | | 0.196 | 0.131 | 1.36E-01 | 9.81E-01 |
| LPA C18:0 | -0.110 | 0.109 | 3.16E-01 | 8.89E-01 | -0.048 | 0.107 | 6.52E-01 | 9.94E-01 | | -0.044 | 0.105 | 6.78E-01 | 9.81E-01 |
| cLPA C20:4 | 0.085 | 0.086 | 3.25E-01 | 8.89E-01 | -0.028 | 0.085 | 7.40E-01 | 9.94E-01 | | 0.002 | 0.083 | 9.81E-01 | 9.81E-01 |
| LPA C14:0 | -0.078 | 0.096 | 4.14E-01 | 8.89E-01 | -0.006 | 0.094 | 9.45E-01 | 9.94E-01 | | 0.016 | 0.092 | 8.64E-01 | 9.81E-01 |
| LPA C20:5 | -0.086 | 0.114 | 4.51E-01 | 8.89E-01 | 0.047 | 0.112 | 6.73E-01 | 9.94E-01 | | 0.083 | 0.110 | 4.54E-01 | 9.81E-01 |
| cLPA C18:2 | 0.074 | 0.102 | 4.71E-01 | 8.89E-01 | -0.060 | 0.100 | 5.49E-01 | 9.94E-01 | | -0.042 | 0.098 | 6.74E-01 | 9.81E-01 |
| LPA C16:0 | -0.066 | 0.104 | 5.30E-01 | 9.01E-01 | -0.039 | 0.102 | 7.01E-01 | 9.94E-01 | | -0.040 | 0.100 | 6.88E-01 | 9.81E-01 |
| LPA C18:2 | 0.048 | 0.122 | 6.92E-01 | 9.38E-01 | -0.141 | 0.119 | 2.39E-01 | 8.12E-01 | | -0.066 | 0.117 | 5.72E-01 | 9.81E-01 |
| LPA C22:4 | 0.050 | 0.134 | 7.07E-01 | 9.38E-01 | -0.079 | 0.132 | 5.52E-01 | 9.94E-01 | | -0.052 | 0.129 | 6.87E-01 | 9.81E-01 |
| LPA C16:1 | -0.037 | 0.101 | 7.17E-01 | 9.38E-01 | 0.098 | 0.099 | 3.25E-01 | 9.20E-01 | | 0.082 | 0.097 | 4.01E-01 | 9.81E-01 |
| LPA C18:3 | -0.020 | 0.114 | 8.64E-01 | 9.91E-01 | 0.001 | 0.111 | 9.94E-01 | 9.94E-01 | | 0.007 | 0.109 | 9.51E-01 | 9.81E-01 |
| LPA C18:1 | -0.017 | 0.130 | 8.99E-01 | 9.91E-01 | -0.039 | 0.128 | 7.63E-01 | 9.94E-01 | | 0.003 | 0.125 | 9.79E-01 | 9.81E-01 |
| cLPA C18:1 | 0.011 | 0.130 | 9.33E-01 | 9.91E-01 | 0.169 | 0.127 | 1.86E-01 | 8.12E-01 | | 0.107 | 0.125 | 3.92E-01 | 9.81E-01 |
| cLPA C18:0 | 0.000 | 0.117 | 9.99E-01 | 9.99E-01 | -0.006 | 0.114 | 9.57E-01 | 9.94E-01 | | -0.010 | 0.110 | 9.30E-01 | 9.81E-01 |

Abbreviations: β, regression coefficient; SE, standard error; FDR, false discovery rate; LPA, lysophosphatidic acid; cLPA, cyclic lysophosphatidic acid

**Supplementary Table 3: Association of metabolites in cerebrospinal fluid with *APOE* 22/23 versus *APOE* 33**

|  | **ACE cohort** | | | **Heidelberg/Mannheim samples** | | | **Meta-analysis** | | | | |
| --- | --- | --- | --- | --- | --- | --- | --- | --- | --- | --- | --- |
|  | **β** | **SE** | ***P-value*** | **β** | **SE** | ***P-value*** | **β** | **SE** | **Direction** | ***P-value*** | ***FDR*** |
| LPA C16:0 | 0.332 | 0.323 | 3.07E-01 | -0.326 | 0.358 | 3.76E-01 | 0.037 | 0.240 | +- | 8.78E-01 | 9.26E-01 |
| LPA C18:0 | 0.290 | 0.307 | 3.48E-01 | 0.006 | 0.416 | 9.88E-01 | 0.190 | 0.247 | ++ | 4.43E-01 | 9.26E-01 |
| LPA C20:5 | -0.265 | 0.283 | 3.51E-01 | -0.610 | 0.510 | 2.50E-01 | -0.346 | 0.247 | -- | 1.62E-01 | 9.26E-01 |
| cLPA C18:0 | 0.246 | 0.315 | 4.37E-01 | -0.546 | 0.435 | 2.28E-01 | -0.027 | 0.255 | +- | 9.17E-01 | 9.26E-01 |
| LPA C20:4 | 0.201 | 0.277 | 4.71E-01 | -0.492 | 0.651 | 4.61E-01 | 0.095 | 0.255 | +- | 7.10E-01 | 9.26E-01 |
| LPA C18:2 | 0.237 | 0.331 | 4.75E-01 | -1.080 | 0.465 | 3.36E-02 | -0.206 | 0.269 | +- | 4.45E-01 | 9.26E-01 |
| LPA C18:1 | 0.182 | 0.278 | 5.14E-01 | -0.490 | 0.498 | 3.40E-01 | 0.023 | 0.243 | +- | 9.26E-01 | 9.26E-01 |
| LPA C22:4 | 0.194 | 0.300 | 5.19E-01 | -0.893 | 0.465 | 7.26E-02 | -0.126 | 0.252 | +- | 6.18E-01 | 9.26E-01 |
| LPA C20:1 | -0.181 | 0.294 | 5.40E-01 | -1.275 | 0.542 | 3.19E-02 | -0.430 | 0.259 | -- | 9.66E-02 | 9.17E-01 |
| aLPA C16:1 | 0.095 | 0.206 | 6.46E-01 | -0.596 | 0.465 | 2.18E-01 | -0.019 | 0.188 | +- | 9.22E-01 | 9.26E-01 |
| cLPA C18:1 | -0.106 | 0.285 | 7.12E-01 | -1.499 | 0.364 | 8.09E-04 | -0.636 | 0.225 | -- | 4.66E-03 | 8.85E-02 |
| LPA C22:5 | 0.077 | 0.317 | 8.10E-01 | -0.847 | 0.513 | 1.18E-01 | -0.179 | 0.270 | +- | 5.08E-01 | 9.26E-01 |
| LPA C14:0 | 0.057 | 0.291 | 8.44E-01 | -0.740 | 0.546 | 1.95E-01 | -0.118 | 0.257 | +- | 6.45E-01 | 9.26E-01 |
| aLPA C18:1 | -0.042 | 0.223 | 8.52E-01 | -0.278 | 0.432 | 5.29E-01 | -0.091 | 0.198 | -- | 6.44E-01 | 9.26E-01 |
| LPA C22:6 | -0.048 | 0.336 | 8.88E-01 | -0.265 | 0.589 | 6.59E-01 | -0.101 | 0.292 | -- | 7.30E-01 | 9.26E-01 |
| cLPA C16:0 | -0.034 | 0.244 | 8.89E-01 | -0.082 | 0.612 | 8.95E-01 | -0.041 | 0.227 | -- | 8.58E-01 | 9.26E-01 |
| LPA C16:1 | 0.040 | 0.308 | 8.96E-01 | -0.892 | 0.610 | 1.63E-01 | -0.149 | 0.275 | +- | 5.89E-01 | 9.26E-01 |
| Isomer-LPA C22:5 | 0.026 | 0.316 | 9.36E-01 | -0.484 | 0.556 | 3.97E-01 | -0.099 | 0.275 | +- | 7.19E-01 | 9.26E-01 |
| LPA C20:3 | -0.002 | 0.253 | 9.95E-01 | -0.287 | 0.443 | 5.27E-01 | -0.072 | 0.220 | -- | 7.44E-01 | 9.26E-01 |

Abbreviations: APOE, Apolipoprotein E; β, regression coefficient; SE, standard error; FDR, false discovery rate; LPA, lysophosphatidic acid; cLPA, cyclic lysophosphatidic acid; aLPA, alkyl-Lysophosphatidic acid

**Note:** Direction column indicates the direction of regression co-efficient of association in the ACE and Heidelberg/Mannheim cohort respectively

**Supplementary Table 4: Association of metabolites in cerebrospinal fluid with *APOE* 44/34/24 vs *APOE* 33**

|  | **ACE cohort** | | | **Heidelberg/Mannheim samples** | | | **Meta-analysis** | | | | |
| --- | --- | --- | --- | --- | --- | --- | --- | --- | --- | --- | --- |
|  | **β** | **SE** | ***P-value*** | **β** | **SE** | ***P-value*** | **β** | **SE** | **Direction** | ***P-value*** | ***FDR*** |
| LPA C14:0 | 0.229 | 0.166 | 1.70E-01 | -0.115 | 0.337 | 7.34E-01 | 0.162 | 0.149 | +- | 2.77E-01 | 8.92E-01 |
| LPA C18:0 | 0.157 | 0.170 | 3.59E-01 | -0.169 | 0.342 | 6.26E-01 | 0.092 | 0.152 | +- | 5.45E-01 | 8.92E-01 |
| cLPA C18:0 | 0.152 | 0.169 | 3.68E-01 | -0.010 | 0.316 | 9.75E-01 | 0.116 | 0.149 | +- | 4.34E-01 | 8.92E-01 |
| LPA C20:4 | -0.141 | 0.157 | 3.72E-01 | 0.247 | 0.351 | 4.86E-01 | -0.076 | 0.144 | -+ | 5.98E-01 | 8.92E-01 |
| LPA C16:1 | 0.154 | 0.173 | 3.74E-01 | -0.176 | 0.336 | 6.04E-01 | 0.085 | 0.154 | +- | 5.80E-01 | 8.92E-01 |
| LPA C20:5 | -0.149 | 0.174 | 3.93E-01 | -0.062 | 0.335 | 8.53E-01 | -0.131 | 0.154 | -- | 3.97E-01 | 8.92E-01 |
| LPA C20:3 | -0.127 | 0.149 | 3.94E-01 | 0.568 | 0.351 | 1.16E-01 | -0.021 | 0.137 | -+ | 8.77E-01 | 9.25E-01 |
| cLPA C16:0 | -0.106 | 0.145 | 4.66E-01 | -0.049 | 0.319 | 8.79E-01 | -0.096 | 0.132 | -- | 4.66E-01 | 8.92E-01 |
| aLPA C18:1 | 0.058 | 0.112 | 6.07E-01 | 0.648 | 0.330 | 5.87E-02 | 0.119 | 0.106 | ++ | 2.63E-01 | 8.92E-01 |
| LPA C18:2 | -0.089 | 0.184 | 6.27E-01 | 0.089 | 0.354 | 8.04E-01 | -0.052 | 0.163 | -+ | 7.51E-01 | 8.92E-01 |
| LPA C22:6 | 0.079 | 0.178 | 6.59E-01 | -0.458 | 0.307 | 1.46E-01 | -0.056 | 0.154 | +- | 7.16E-01 | 8.92E-01 |
| aLPA C16:1 | 0.050 | 0.114 | 6.62E-01 | 0.292 | 0.358 | 4.21E-01 | 0.072 | 0.109 | ++ | 5.06E-01 | 8.92E-01 |
| Isomer-LPA C22:5 | -0.077 | 0.176 | 6.64E-01 | 0.210 | 0.329 | 5.29E-01 | -0.013 | 0.155 | -+ | 9.34E-01 | 9.34E-01 |
| LPA C18:1 | -0.054 | 0.151 | 7.21E-01 | -0.113 | 0.296 | 7.06E-01 | -0.066 | 0.134 | -- | 6.23E-01 | 8.92E-01 |
| LPA C20:1 | -0.057 | 0.165 | 7.29E-01 | -0.088 | 0.324 | 7.88E-01 | -0.064 | 0.147 | -- | 6.66E-01 | 8.92E-01 |
| LPA C16:0 | 0.048 | 0.186 | 7.98E-01 | 0.101 | 0.286 | 7.26E-01 | 0.064 | 0.156 | ++ | 6.84E-01 | 8.92E-01 |
| LPA C22:4 | -0.043 | 0.186 | 8.16E-01 | -0.210 | 0.306 | 4.98E-01 | -0.088 | 0.159 | -- | 5.79E-01 | 8.92E-01 |
| LPA C22:5 | -0.018 | 0.178 | 9.19E-01 | -0.076 | 0.335 | 8.23E-01 | -0.031 | 0.157 | -- | 8.45E-01 | 9.25E-01 |
| cLPA C18:1 | -0.017 | 0.164 | 9.20E-01 | -0.496 | 0.298 | 1.06E-01 | -0.128 | 0.144 | -- | 3.72E-01 | 8.92E-01 |

Abbreviations: APOE, Apolipoprotein E; β, regression coefficient; SE, standard error; FDR, false discovery rate; LPA, lysophosphatidic acid; cLPA, cyclic lysophosphatidic acid; aLPA, alkyl-Lysophosphatidic acid

**Note:** Direction column indicates the direction of regression co-efficient of association in the ACE and Heidelberg/Mannheim cohort respectively

**Supplementary Table 5: Association of metabolites measured in CSF with MCI to AD conversion in ACE cohort**

|  | **β** | **SE** | ***P-value*** | ***FDR*** |
| --- | --- | --- | --- | --- |
| LPA C16:1 | -0.472 | 0.160 | 3.25E-03 | 4.41E-02 |
| LPA C16:0 | -0.412 | 0.145 | 4.64E-03 | 4.41E-02 |
| LPA C14:0 | -0.362 | 0.168 | 3.13E-02 | 1.29E-01 |
| LPA C18:2 | -0.386 | 0.184 | 3.60E-02 | 1.29E-01 |
| LPA C22:5 | -0.385 | 0.187 | 3.94E-02 | 1.29E-01 |
| LPA C20:3 | -0.413 | 0.203 | 4.20E-02 | 1.29E-01 |
| LPA C18:1 | -0.410 | 0.207 | 4.75E-02 | 1.29E-01 |
| LPA C22:6 | -0.323 | 0.173 | 6.16E-02 | 1.46E-01 |
| LPA C20:4 | -0.398 | 0.221 | 7.18E-02 | 1.51E-01 |
| LPA C18:0 | -0.293 | 0.169 | 8.35E-02 | 1.59E-01 |
| cLPA C18:1 | -0.294 | 0.183 | 1.09E-01 | 1.89E-01 |
| aLPA C16:1 | -0.306 | 0.237 | 1.98E-01 | 3.13E-01 |
| Isomer-LPA C22:5 | -0.174 | 0.171 | 3.09E-01 | 4.35E-01 |
| cLPA C18:0 | -0.178 | 0.179 | 3.20E-01 | 4.35E-01 |
| aLPA C18:1 | -0.239 | 0.258 | 3.54E-01 | 4.49E-01 |
| cLPA C16:0 | -0.163 | 0.189 | 3.88E-01 | 4.54E-01 |
| LPA C20:1 | -0.149 | 0.180 | 4.06E-01 | 4.54E-01 |
| LPA C22:4 | -0.037 | 0.166 | 8.23E-01 | 8.69E-01 |
| LPA C20:5 | -0.015 | 0.187 | 9.35E-01 | 9.35E-01 |

Abbreviations: β, regression coefficient; SE, standard error; FDR, false discovery rate; LPA, lysophosphatidic acid; cLPA, cyclic lysophosphatidic acid; aLPA, alkyl-Lysophosphatidic acid

**Supplementary Table 6: Association of metabolites measured in CSF with MCI to AD conversion adjusting for *APOE* in ACE cohort**

|  | **β** | **SE** | ***P-value*** | ***FDR*** |
| --- | --- | --- | --- | --- |
| LPA C16:1 | -0.473 | 0.160 | 3.23E-03 | 4.84E-02 |
| LPA C16:0 | -0.409 | 0.146 | 5.10E-03 | 4.84E-02 |
| LPA C14:0 | -0.370 | 0.169 | 2.83E-02 | 1.43E-01 |
| LPA C18:2 | -0.382 | 0.185 | 3.92E-02 | 1.43E-01 |
| LPA C22:5 | -0.385 | 0.188 | 4.05E-02 | 1.43E-01 |
| LPA C20:3 | -0.407 | 0.204 | 4.64E-02 | 1.43E-01 |
| LPA C18:1 | -0.405 | 0.209 | 5.28E-02 | 1.43E-01 |
| LPA C22:6 | -0.328 | 0.174 | 6.02E-02 | 1.43E-01 |
| LPA C20:4 | -0.395 | 0.227 | 8.23E-02 | 1.67E-01 |
| LPA C18:0 | -0.290 | 0.170 | 8.80E-02 | 1.67E-01 |
| cLPA C18:1 | -0.294 | 0.184 | 1.10E-01 | 1.90E-01 |
| aLPA C16:1 | -0.299 | 0.238 | 2.10E-01 | 3.32E-01 |
| Isomer-LPA C22:5 | -0.174 | 0.173 | 3.16E-01 | 4.61E-01 |
| cLPA C18:0 | -0.172 | 0.181 | 3.42E-01 | 4.64E-01 |
| aLPA C18:1 | -0.230 | 0.262 | 3.79E-01 | 4.79E-01 |
| cLPA C16:0 | -0.157 | 0.191 | 4.11E-01 | 4.79E-01 |
| LPA C20:1 | -0.144 | 0.181 | 4.28E-01 | 4.79E-01 |
| LPA C22:4 | -0.031 | 0.166 | 8.54E-01 | 8.82E-01 |
| LPA C20:5 | -0.028 | 0.191 | 8.82E-01 | 8.82E-01 |

Abbreviations: β, regression coefficient; SE, standard error; FDR, false discovery rate; LPA, lysophosphatidic acid; cLPA, cyclic lysophosphatidic acid; aLPA, alkyl-Lysophosphatidic acid

|  | **Aβ measures in model** | | | | **LPA measures in model** | | | |
| --- | --- | --- | --- | --- | --- | --- | --- | --- |
|  | **β** | **SE** | ***P-value*** | ***FDR*** | **β** | **SE** | ***P-value*** | ***FDR*** |
| aLPA C16:1 | -1.080 | 0.230 | 2.53E-06 | 6.87E-06 | -0.110 | 0.228 | 6.29E-01 | 7.96E-01 |
| aLPA C18:1 | -1.109 | 0.232 | 1.78E-06 | 6.87E-06 | 0.124 | 0.258 | 6.31E-01 | 7.96E-01 |
| cLPA C16:0 | -1.183 | 0.250 | 2.21E-06 | 6.87E-06 | 0.225 | 0.220 | 3.05E-01 | 7.96E-01 |
| cLPA C18:0 | -1.119 | 0.237 | 2.37E-06 | 6.87E-06 | 0.102 | 0.199 | 6.08E-01 | 7.96E-01 |
| LPA C20:1 | -1.134 | 0.240 | 2.34E-06 | 6.87E-06 | 0.133 | 0.192 | 4.87E-01 | 7.96E-01 |
| LPA C20:5 | -1.110 | 0.229 | 1.26E-06 | 6.87E-06 | -0.192 | 0.216 | 3.74E-01 | 7.96E-01 |
| LPA C22:4 | -1.197 | 0.252 | 1.94E-06 | 6.87E-06 | 0.236 | 0.185 | 2.01E-01 | 7.96E-01 |
| Isomer-LPA C22:5 | -1.082 | 0.231 | 2.93E-06 | 6.96E-06 | -0.012 | 0.188 | 9.47E-01 | 9.47E-01 |
| LPA C18:0 | -1.062 | 0.231 | 4.36E-06 | 8.45E-06 | -0.095 | 0.170 | 5.74E-01 | 7.96E-01 |
| LPA C22:5 | -1.027 | 0.224 | 4.45E-06 | 8.45E-06 | -0.294 | 0.213 | 1.67E-01 | 7.96E-01 |
| cLPA C18:1 | -1.056 | 0.233 | 5.83E-06 | 1.00E-05 | -0.094 | 0.206 | 6.47E-01 | 7.96E-01 |
| LPA C14:0 | -1.042 | 0.231 | 6.53E-06 | 1.00E-05 | -0.160 | 0.169 | 3.45E-01 | 7.96E-01 |
| LPA C18:1 | -1.068 | 0.240 | 8.32E-06 | 1.00E-05 | -0.047 | 0.221 | 8.30E-01 | 9.28E-01 |
| LPA C20:3 | -1.038 | 0.233 | 8.44E-06 | 1.00E-05 | -0.180 | 0.229 | 4.33E-01 | 7.96E-01 |
| LPA C20:4 | -1.075 | 0.240 | 7.27E-06 | 1.00E-05 | -0.028 | 0.226 | 9.01E-01 | 9.47E-01 |
| LPA C22:6 | -1.054 | 0.236 | 8.04E-06 | 1.00E-05 | -0.077 | 0.181 | 6.70E-01 | 7.96E-01 |
| LPA C18:2 | -1.040 | 0.236 | 1.07E-05 | 1.20E-05 | -0.124 | 0.195 | 5.25E-01 | 7.96E-01 |
| LPA C16:0 | -1.011 | 0.234 | 1.64E-05 | 1.73E-05 | -0.209 | 0.153 | 1.73E-01 | 7.96E-01 |
| LPA C16:1 | -0.982 | 0.234 | 2.67E-05 | 2.67E-05 | -0.239 | 0.175 | 1.72E-01 | 7.96E-01 |

**Supplementary Table 7: Association of metabolites measured in CSF with MCI to AD conversion adjusted for amyloid beta 42 levels in ACE cohort**

Abbreviations: β, regression coefficient; SE, standard error; FDR, false discovery rate; LPA, lysophosphatidic acid; cLPA, cyclic lysophosphatidic acid; aLPA, alkyl-Lysophosphatidic acid

**Supplementary Table 8: Association of metabolites measured in plasma with MCI to AD conversion in ACE cohort**

|  | **β** | **SE** | ***P-value*** | ***FDR*** |
| --- | --- | --- | --- | --- |
| LPA C20:1 | 0.599 | 0.254 | 1.84E-02 | 3.12E-01 |
| Isomer-LPA C22:5 | 0.284 | 0.177 | 1.08E-01 | 3.52E-01 |
| cLPA C20:4 | -0.265 | 0.167 | 1.13E-01 | 3.52E-01 |
| LPA C16:1 | 0.334 | 0.222 | 1.33E-01 | 3.52E-01 |
| cLPA C18:0 | -0.295 | 0.199 | 1.38E-01 | 3.52E-01 |
| LPA C14:0 | 0.302 | 0.204 | 1.38E-01 | 3.52E-01 |
| cLPA C16:0 | -0.324 | 0.233 | 1.65E-01 | 3.52E-01 |
| LPA C18:2 | -0.313 | 0.237 | 1.85E-01 | 3.52E-01 |
| LPA C20:5 | -0.306 | 0.232 | 1.87E-01 | 3.52E-01 |
| LPA C20:3 | -0.338 | 0.277 | 2.23E-01 | 3.79E-01 |
| LPA C18:1 | -0.265 | 0.253 | 2.95E-01 | 4.28E-01 |
| cLPA C18:2 | -0.214 | 0.207 | 3.02E-01 | 4.28E-01 |
| LPA C18:3 | 0.220 | 0.229 | 3.37E-01 | 4.40E-01 |
| cLPA C18:1 | -0.155 | 0.255 | 5.42E-01 | 6.59E-01 |
| LPA C22:4 | -0.133 | 0.255 | 6.02E-01 | 6.82E-01 |
| LPA C18:0 | -0.095 | 0.216 | 6.61E-01 | 7.02E-01 |
| LPA C16:0 | 0.017 | 0.195 | 9.31E-01 | 9.31E-01 |

Abbreviations: β, regression coefficient; SE, standard error; FDR, false discovery rate; LPA, lysophosphatidic acid; cLPA, cyclic lysophosphatidic acid

**Supplementary Table 9: Association of metabolites measured in CSF with MCI to AD conversion in Heidelberg/Mannheim sample**

|  | **β** | **SE** | ***P-value*** | ***FDR*** |
| --- | --- | --- | --- | --- |
| LPA C22:5 | 0.906 | 0.351 | 9.85E-03 | 1.40E-01 |
| LPA C14:0 | 0.618 | 0.272 | 2.30E-02 | 1.40E-01 |
| cLPA C16:0 | 0.633 | 0.282 | 2.48E-02 | 1.40E-01 |
| LPA483 | 0.652 | 0.308 | 3.44E-02 | 1.40E-01 |
| LPA C20:3 | 0.512 | 0.254 | 4.36E-02 | 1.40E-01 |
| LPA C18:1 | 0.685 | 0.345 | 4.69E-02 | 1.40E-01 |
| LPA C20:5 | 0.563 | 0.289 | 5.16E-02 | 1.40E-01 |
| LPA C16:1 | 0.457 | 0.269 | 8.91E-02 | 2.02E-01 |
| LPA C20:4 | 0.459 | 0.275 | 9.57E-02 | 2.02E-01 |
| LPA C16:0 | 0.431 | 0.333 | 1.96E-01 | 3.72E-01 |
| cLPA C18:0 | 0.341 | 0.281 | 2.25E-01 | 3.89E-01 |
| aLPA C18:1 | 0.314 | 0.284 | 2.70E-01 | 4.28E-01 |
| LPA C22:4 | 0.237 | 0.278 | 3.93E-01 | 5.75E-01 |
| LPA C20:1 | 0.190 | 0.248 | 4.44E-01 | 5.78E-01 |
| LPA C18:2 | 0.230 | 0.309 | 4.56E-01 | 5.78E-01 |
| LPA C18:0 | 0.169 | 0.269 | 5.29E-01 | 6.05E-01 |
| aLPA C16:1 | 0.154 | 0.252 | 5.42E-01 | 6.05E-01 |
| cLPA C18:1 | 0.101 | 0.284 | 7.22E-01 | 7.59E-01 |
| LPA C22:6 | 0.096 | 0.313 | 7.59E-01 | 7.59E-01 |

Abbreviations: β, regression coefficient; SE, standard error; FDR, false discovery rate; LPA, lysophosphatidic acid; cLPA, cyclic lysophosphatidic acid; aLPA, alkyl-Lysophosphatidic acid

**Supplementary Table 10:** Association of the Mini-Mental State Examination (MMSE) with LPAs in CSF

|  | **ACE cohort** | | | **Heidelberg/Mannheim samples** | | | **Meta-analysis** | | | | |
| --- | --- | --- | --- | --- | --- | --- | --- | --- | --- | --- | --- |
|  | **β** | **SE** | ***P-value*** | **β** | **SE** | ***P-value*** | **β** | **SE** | **Direction** | ***P-value*** | ***FDR*** |
| aLPA C16:1 | 0.253 | 0.1218 | 4.00E-02 | -0.227 | 0.166 | 1.80E-01 | 0.0847 | 0.0982 | +- | 3.89E-01 | 8.51E-01 |
| aLPA C18:1 | 0.155 | 0.120302 | 1.99E-01 | -0.304 | 0.172 | 8.62E-02 | 0.0043 | 0.0986 | +- | 9.65E-01 | 9.65E-01 |
| cLPA C16:0 | 0.061 | 0.098411 | 5.36E-01 | 0.087 | 0.191 | 6.53E-01 | 0.0664 | 0.0875 | ++ | 4.48E-01 | 8.51E-01 |
| cLPA C18:0 | 0.085 | 0.084596 | 3.15E-01 | -0.200 | 0.193 | 3.09E-01 | 0.0396 | 0.0775 | +- | 6.10E-01 | 9.45E-01 |
| cLPA C18:1 | 0.098 | 0.087199 | 2.62E-01 | -0.001 | 0.189 | 9.95E-01 | 0.0807 | 0.0792 | +- | 3.08E-01 | 8.51E-01 |
| LPA C14:0 | 0.143 | 0.086015 | 9.97E-02 | 0.022 | 0.183 | 9.04E-01 | 0.1208 | 0.0778 | ++ | 1.21E-01 | 7.64E-01 |
| LPA C16:0 | 0.062 | 0.077919 | 4.28E-01 | -0.261 | 0.210 | 2.23E-01 | 0.023 | 0.0731 | +- | 7.53E-01 | 9.45E-01 |
| LPA C16:1 | 0.098 | 0.083006 | 2.40E-01 | -0.072 | 0.178 | 6.88E-01 | 0.0677 | 0.0753 | +- | 3.69E-01 | 8.51E-01 |
| LPA C18:0 | 0.049 | 0.084276 | 5.60E-01 | -0.160 | 0.181 | 3.84E-01 | 0.0122 | 0.0764 | +- | 8.74E-01 | 9.65E-01 |
| LPA C18:1 | 0.095 | 0.095301 | 3.23E-01 | -0.065 | 0.208 | 7.58E-01 | 0.067 | 0.0867 | +- | 4.40E-01 | 8.51E-01 |
| LPA C18:2 | 0.043 | 0.078775 | 5.87E-01 | -0.028 | 0.171 | 8.72E-01 | 0.0305 | 0.0715 | +- | 6.70E-01 | 9.45E-01 |
| LPA C20:1 | 0.094 | 0.086067 | 2.76E-01 | 0.134 | 0.177 | 4.53E-01 | 0.1019 | 0.0774 | ++ | 1.88E-01 | 8.51E-01 |
| LPA C20:3 | 0.071 | 0.095647 | 4.60E-01 | -0.302 | 0.163 | 7.29E-02 | -0.0244 | 0.0825 | +- | 7.67E-01 | 9.45E-01 |
| LPA C20:4 | 0.034 | 0.090555 | 7.08E-01 | 0.007 | 0.172 | 9.66E-01 | 0.0282 | 0.0801 | ++ | 7.25E-01 | 9.45E-01 |
| LPA C20:5 | 0.106 | 0.083301 | 2.04E-01 | -0.163 | 0.188 | 3.92E-01 | 0.0621 | 0.0761 | +- | 4.15E-01 | 8.51E-01 |
| LPA C22:4 | 0.012 | 0.078655 | 8.82E-01 | 0.064 | 0.197 | 7.47E-01 | 0.0189 | 0.073 | ++ | 7.96E-01 | 9.45E-01 |
| LPA C22:5 | 0.010 | 0.082065 | 9.03E-01 | -0.025 | 0.187 | 8.96E-01 | 0.0044 | 0.0752 | +- | 9.53E-01 | 9.65E-01 |
| LPA C22:6 | 0.131 | 0.08059 | 1.07E-01 | 0.193 | 0.193 | 3.26E-01 | 0.14 | 0.0744 | ++ | 5.98E-02 | 7.64E-01 |
| Isomer-LPA C22:5 | 0.125 | 0.080508 | 1.23E-01 | 0.067 | 0.181 | 7.15E-01 | 0.1153 | 0.0736 | ++ | 1.17E-01 | 7.64E-01 |

**Abbreviations:** LPA, lysophosphatidic acid; cLPA, cyclic lysophosphatidic acid; SE, standard error; *FDR*, false discovery rate

Note: Direction column indicates the direction of regression co-efficient of association in the ACE and Heidelberg/Mannheim cohort respectively

**Supplementary Table 11:** Association of the clinical dementia score (CDR) with LPAs in CSF

|  | **ACE cohort** | | | **Heidelberg/Mannheim samples** | | | **Meta-analysis** | | | | |
| --- | --- | --- | --- | --- | --- | --- | --- | --- | --- | --- | --- |
|  | **β** | **SE** | ***P-value*** | **β** | **SE** | ***P-value*** | **β** | **SE** | **Direction** | ***P-value*** | ***FDR*** |
| aLPA C16:1 | -0.001 | 0.044 | 9.75E-01 | 0.254 | 0.087 | 6.01E-03 | 0.051 | 0.039 | -+ | 1.94E-01 | 9.71E-01 |
| aLPA C18:1 | 0.038 | 0.043 | 3.79E-01 | 0.229 | 0.094 | 2.09E-02 | 0.071 | 0.039 | ++ | 7.06E-02 | 9.71E-01 |
| cLPA C16:0 | -0.019 | 0.035 | 5.86E-01 | -0.144 | 0.106 | 1.84E-01 | -0.031 | 0.033 | -- | 3.47E-01 | 9.71E-01 |
| cLPA C18:0 | -0.015 | 0.030 | 6.09E-01 | 0.062 | 0.111 | 5.84E-01 | -0.010 | 0.029 | -+ | 7.25E-01 | 9.71E-01 |
| cLPA C18:1 | -0.003 | 0.031 | 9.20E-01 | 0.074 | 0.107 | 4.91E-01 | 0.003 | 0.030 | -+ | 9.22E-01 | 1.00E+00 |
| LPA C14:0 | -0.001 | 0.031 | 9.74E-01 | -0.095 | 0.103 | 3.62E-01 | -0.009 | 0.030 | -- | 7.67E-01 | 9.71E-01 |
| LPA C16:0 | -0.001 | 0.028 | 9.58E-01 | 0.028 | 0.122 | 8.19E-01 | 0.000 | 0.027 | -+ | 1.00E+00 | 1.00E+00 |
| LPA C16:1 | 0.002 | 0.030 | 9.44E-01 | -0.043 | 0.101 | 6.72E-01 | -0.002 | 0.028 | +- | 9.59E-01 | 1.00E+00 |
| LPA C18:0 | -0.013 | 0.030 | 6.57E-01 | 0.017 | 0.104 | 8.75E-01 | -0.011 | 0.029 | -+ | 7.01E-01 | 9.71E-01 |
| LPA C18:1 | 0.001 | 0.034 | 9.67E-01 | -0.073 | 0.118 | 5.41E-01 | -0.004 | 0.033 | +- | 8.97E-01 | 1.00E+00 |
| LPA C18:2 | 0.013 | 0.028 | 6.45E-01 | 0.013 | 0.097 | 8.95E-01 | 0.013 | 0.027 | ++ | 6.31E-01 | 9.71E-01 |
| LPA C20:1 | 0.013 | 0.031 | 6.76E-01 | 0.098 | 0.100 | 3.32E-01 | 0.020 | 0.030 | ++ | 4.90E-01 | 9.71E-01 |
| LPA C20:3 | 0.017 | 0.034 | 6.17E-01 | 0.142 | 0.094 | 1.42E-01 | 0.031 | 0.032 | ++ | 3.26E-01 | 9.71E-01 |
| LPA C20:4 | 0.027 | 0.032 | 3.97E-01 | -0.124 | 0.095 | 2.03E-01 | 0.012 | 0.030 | +- | 6.94E-01 | 9.71E-01 |
| LPA C20:5 | 0.009 | 0.030 | 7.72E-01 | 0.139 | 0.105 | 1.94E-01 | 0.018 | 0.029 | ++ | 5.22E-01 | 9.71E-01 |
| LPA C22:4 | -0.012 | 0.028 | 6.63E-01 | -0.131 | 0.110 | 2.40E-01 | -0.019 | 0.027 | -- | 4.74E-01 | 9.71E-01 |
| LPA C22:5 | 0.035 | 0.029 | 2.22E-01 | -0.078 | 0.106 | 4.64E-01 | 0.028 | 0.028 | +- | 3.23E-01 | 9.71E-01 |
| LPA C22:6 | -0.014 | 0.029 | 6.25E-01 | -0.140 | 0.109 | 2.08E-01 | -0.023 | 0.028 | -- | 4.21E-01 | 9.71E-01 |
| Isomer-LPA C22:5 | -0.041 | 0.029 | 1.55E-01 | 0.007 | 0.103 | 9.49E-01 | -0.038 | 0.028 | -+ | 1.74E-01 | 9.71E-01 |

**Abbreviations:** LPA, lysophosphatidic acid; cLPA, cyclic lysophosphatidic acid; SE, standard error; *FDR*, false discovery rate

**Supplementary Figure 1: Correlation of metabolite levels between plasma and CSF**.


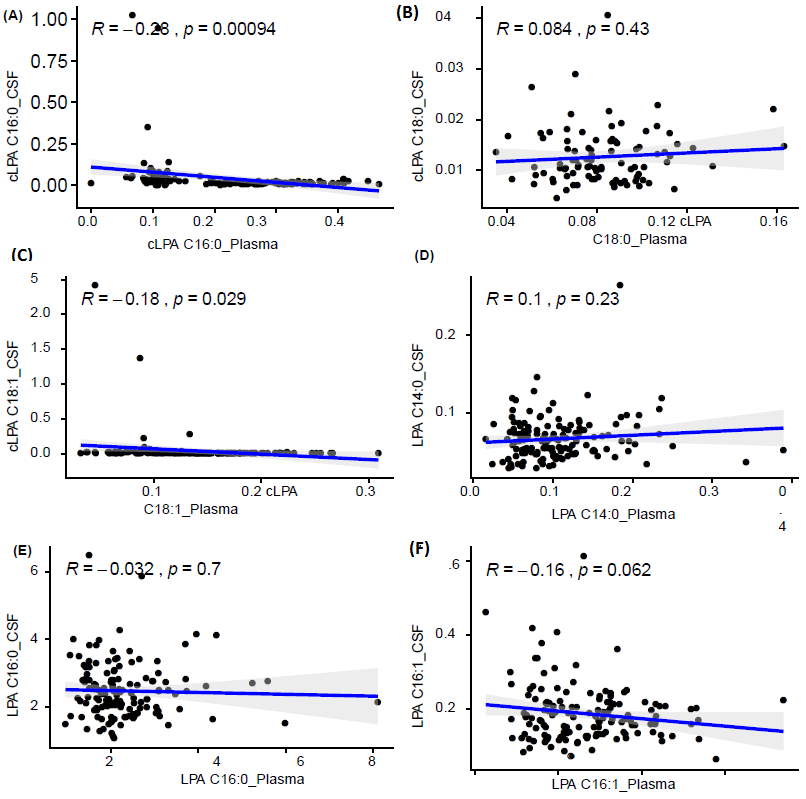


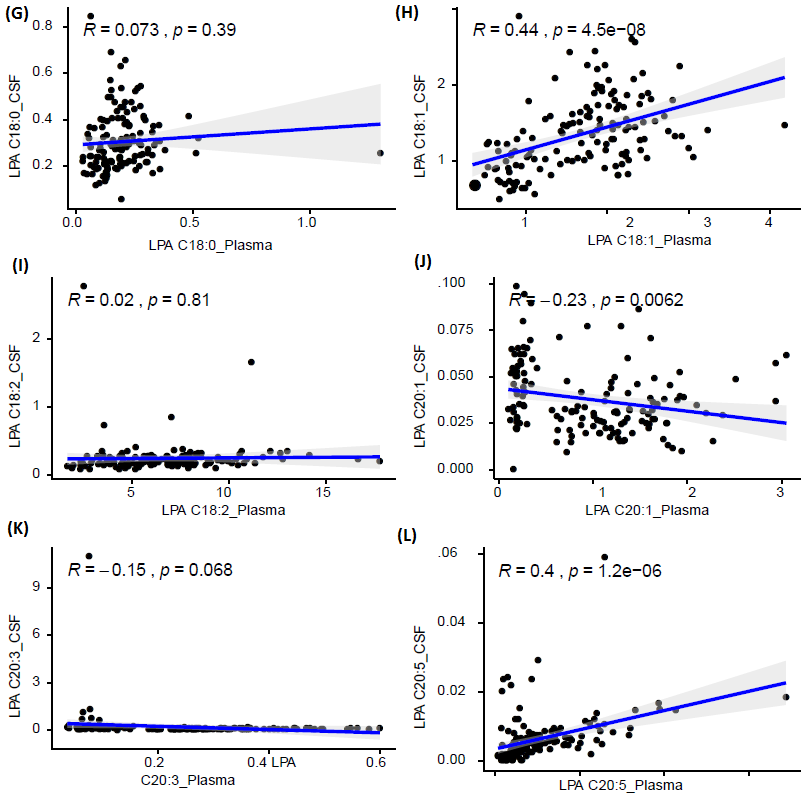


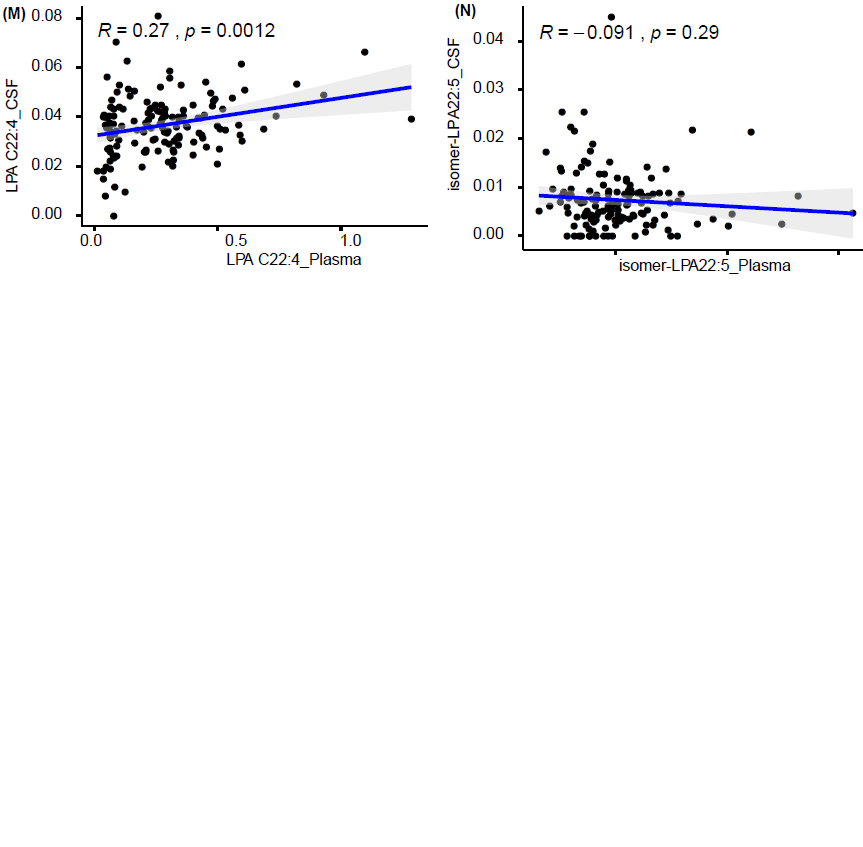


**Supplementary Figure 2: Correlation matrix of CSF LPA metabolites**


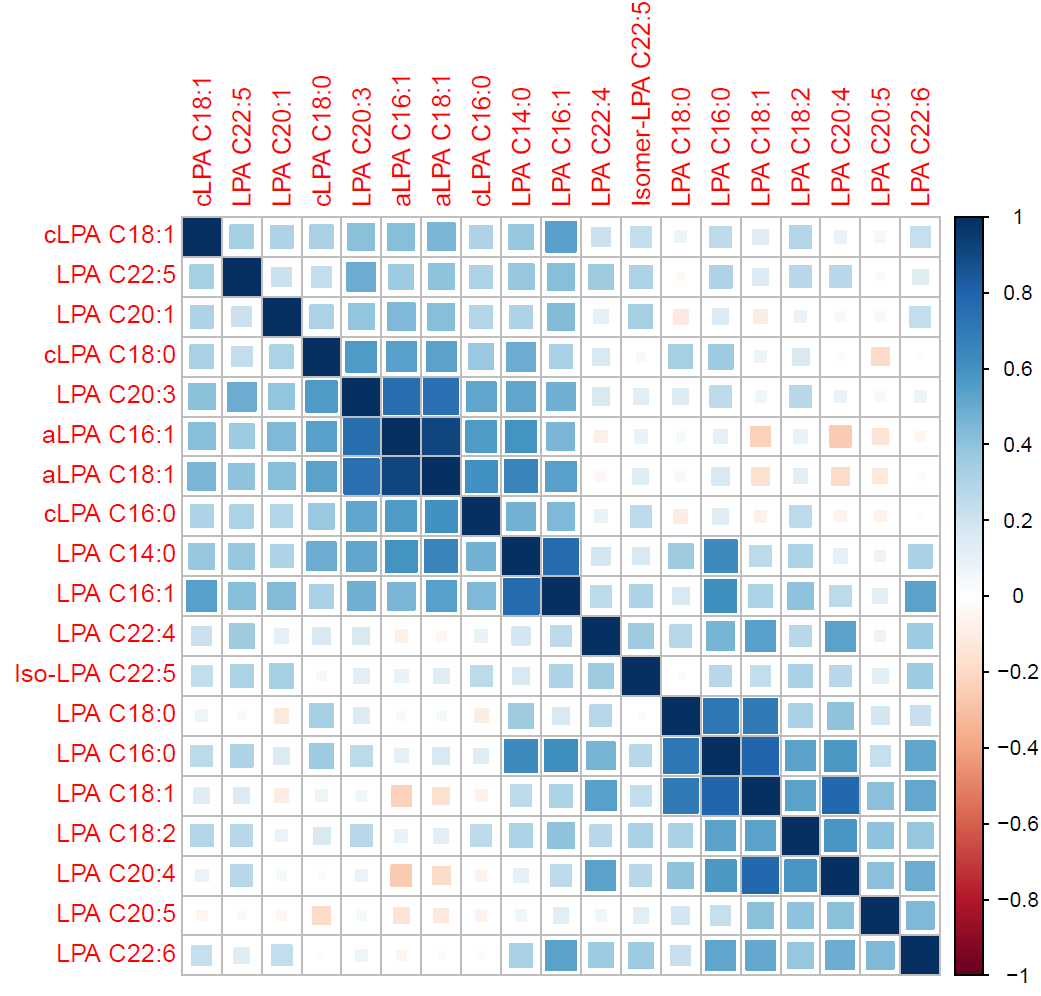

Supplement: Supplementary file 1 — Additional file 1: Table S1. List of detected lysophosphatidic acids in cerebrospinal fluid and plasma. Table S2. Association of metabolites in plasma association with amyloid-beta 42, P-Tau and total tau. Table S3. Association of metabolites in cerebrospinal fluid with APOE 22/23 versus APOE 33. Table S4. Association of metabolites in cerebrospinal fluid with APOE 44/34/24 vs APOE 33. Table S5. Association of metabolites measured in CSF with MCI to AD conversion in ACE cohort. Table S6. Association of metabolites measured in CSF with MCI to AD conversion adjusting for APOE in ACE cohort. Table S7. Association of metabolites measured in CSF with MCI to AD conversion adjusted for amyloid beta 42 levels in ACE cohort. Table S8. Association of metabolites measured in plasma with MCI to AD conversion in ACE cohort. Table S9. Association of metabolites measured in CSF with MCI to AD conversion in Heidelberg/Mannheim sample. Table S10. Association of the Mini-Mental State Examination (MMSE) with LPAs in CSF. Table S11. Association of the clinical dementia score (CDR) with LPAs in CSF. Figure S1. Correlation of metabolite levels between plasma and CSF. Figure S2. Correlation matrix of CSF LPA metabolites. [file 13195_2020_680_MOESM1_ESM.docx]
